# The #chatsafe project. Developing guidelines to help young people communicate safely about suicide on social media: A Delphi study

**DOI:** 10.1371/journal.pone.0206584

**Published:** 2018-11-15

**Authors:** Jo Robinson, Nicole T. M. Hill, Pinar Thorn, Rikki Battersby, Zoe Teh, Nicola J. Reavley, Jane Pirkis, Michelle Lamblin, Simon Rice, Jaelea Skehan

**Affiliations:** 1 Orygen, The National Centre of Excellence in Youth Mental Health, Victoria, Australia; 2 Centre for Youth Mental Health, The University of Melbourne, Victoria, Australia; 3 Centre for Mental Health, The Melbourne School of Population and Global Health, The University of Melbourne, Victoria, Australia; 4 Everymind, Newcastle, New South Wales, Australia; 5 School of Medicine and Public Health, University of Newcastle, Callaghan, New South Wales, Australia; Karolinska Institutet, SWEDEN

## Abstract

**Introduction:**

Many countries have developed guidelines advocating for responsible reporting of suicidal behaviour in traditional media. However, the increasing popularity of social media, particularly among young people, means that complementary guidelines designed to facilitate safe peer-peer communication are required. The aim of this study was to develop a set of evidence informed guidelines to assist young people to communicate about suicide via social media with the input of young people as active participants of the study.

**Methods:**

Systematic searches of the peer-reviewed and grey literature were conducted resulting in a 284-item questionnaire identifying strategies for safe communication about suicide online. The questionnaire was delivered over two rounds to two panels consisting of Australian youth advocates; and international suicide prevention researchers and media and communications specialists. Items were rerated if they were endorsed by 70–79.5% of both panels, or if 80% or more of one panel rated the item as essential or important. All items that were endorsed as essential or important by at least 80% of both panels were included in the final guidelines.

**Results:**

A total of 173 items were included in the final guidelines. These items were organised into the following five sections: 1) Before you post anything online about suicide; 2) Sharing your own thoughts, feelings, or experience with suicidal behaviour online; 3) Communicating about someone you know who is affected by suicidal thoughts, feelings or behaviours; 4) Responding to someone who may be suicidal; 5) Memorial websites, pages and closed groups to honour the deceased.

**Discussion:**

This is the first study to develop a set of evidence-informed guidelines to support young people to talk safely about suicide on social media. It is hoped that they will be a useful resource for young people and those who support them (e.g., parents, teachers, community workers and health professionals).

## Introduction

Suicide is the second-leading cause of death among young people worldwide and rates appear to be increasing [1]. Certain types of media reporting about suicide, such as detailed information about location or methods of suicide, repeated exposure to suicide-related content, and reports that glamourize or sensationalize suicide, have been linked to an increase in suicide deaths [2]. This is thought to be the result of contagion, whereby certain types of reporting may lead to the suicidal behaviour being imitated by others, and young people are thought to be particularly susceptible to this process [3, 4].

For this reason, the role of the media in suicide prevention has long been recognised [1] and many countries, including, but not limited to, Australia, United Kingdom, the United States, Hong Kong, and Sri Lanka have developed media guidelines that advocate for responsible and sensitive reporting and portrayal of suicide [5–9]. International guidelines have also been developed by The World Health Organization, in partnership with the International Association for Suicide Prevention [10]. For the most part, existing guidelines focus on the potential negative impact of aspects of media reporting. Current guidelines also recommend strategies that can be used in media reports to promote positive coping among vulnerable individuals, also known as the Papageno effect [11]. For example, they recommend providing information to local crisis support services; avoiding graphic descriptions of suicide methods, or locations; they caution against glamorizing suicide or using potentially sensationalist language; and they advise against giving too much prominence to stories about suicide, in order to reduce the likelihood of copycat instances as well as distress caused by over-exposure to negative content [5, 6]. Evidence suggests that the implementation of media guidelines can improve the quality of reporting about suicide [12, 13], and when the quality of media reporting improves subsequent reductions in the suicide rate have been observed [14].

The last decade however, has seen the advent of social media, which is particularly popular among young people who use these platforms for a range of purposes, including to communicate about suicide [15, 16]. Some key benefits associated with using social media platforms to communicate about suicide have been identified. These include the accessibility and acceptability of these platforms, the speed via which helpful messages can be transmitted, and the sense of community they provide [15–17]. Conversely, challenges include the potential for the sharing of distressing or sensationalist content, the spreading of information about suicide locations and methods, and the potential for imitative suicidal behaviour [15, 18]. A further challenge that has been identified relates to the lack of evidence-based guidelines to facilitate safe communication about suicide on social media platforms [15]. As a result, consideration needs to be given to how traditional media guidelines can be adapted so that they can be applied to the fast moving and interactive nature of social media, and so that they reflect the ways in which young people use these platforms to talk about suicide [19]. A solution to this might be the development of new guidelines that complement existing ones, but are specifically designed to better equip young people to have safe conversations about suicide via social media platforms.

In developing traditional media guidelines, it has been argued that involvement of journalists from the outset has impacted on their successful uptake by the industry [13, 20]. In our case, the stakeholders we wanted to engage are young people, therefore it made logical sense to include them from the outset. Participatory research design processes are increasingly being recognised as critical to ensure that the views and preferences of end users are accounted for, which in turn is likely to enhance both uptake and engagement [21].

Thus, the aims of this study were to: 1) Develop a set of evidence-informed guidelines to assist young people to communicate safely about suicide via social media; 2) Involve young people as active participants in the study.

## Methods

The study employed the Delphi expert consensus method. This is a technique used to survey the opinions of experts, via a series of questionnaires, in order to establish group consensus about best practice in a particular area when empirical evidence is limited or absent [22]. Statements concerning strategies (e.g., knowledge a person should have, actions a person should take or information or content that should be included) when communicating about suicide online were extracted from peer reviewed and grey literature and incorporated into a 284 item questionnaire that was used to inform the development of a set of guidelines to inform young people on how to communicate safely about suicide online. The current study consisted of five stages: 1) A systematic search of peer reviewed and grey literature; 2) Development of the questionnaire 3) Expert panel formation; 4) Delphi consensus ratings by expert panel members; and 5) Development of the #Chatsafe guidelines. The study was approved by the University of Melbourne Psychology Health and Applied Sciences Human Ethics Sub-committee (HESC 1749618.5).

### Systematic search of the literature

#### Search of the peer reviewed literature

A systematic search of the peer reviewed literature was undertaken to identify strategies for communicating about suicide online. Two authors (NH and PT) conducted initial eligibility screening based on title and abstract, followed by an assessment of full-text versions. We searched English language journals using Medline, PsychINFO, ERIC, and Scopus from January 1, 2000 to September 5, 2017. We chose 2000 as the initial reference period to correspond with the emergence of social media platforms during this time [23]. The following search terms were entered into each database and searched using title, key words, and abstracts: (Suicid* AND (social media OR social network* OR Instagram OR YouTube OR Myspace OR Tumblr OR Snapchat OR Twitter OR Facebook OR Pinterest OR blog* OR chat OR multimedia OR media OR forum OR internet OR online OR web* OR cyber OR net OR technology OR campaign OR messag*) AND (safe* OR policy OR frame* OR prevent* OR monitor OR surveil* OR protect* OR secur* OR guard* OR audit OR guide* OR code OR rule OR protocol OR practice OR instruct* OR recommend* OR report*)).

#### Search of the grey literature

Additionally, we conducted a grey literature search using Google search engines in order to identify lay literature from websites, reports and online brochures. We searched Google, Google Australia, Google New Zealand, Google Canada and Google UK using the search terms identified above. Links from the first 10 pages of each search (representing 100 results) were reviewed, using the title and text underneath. This number of pages was chosen to capture websites that were ranked most relevant, while being a feasible number to screen.

#### Eligibility criteria

Sources from the peer reviewed and grey literature were eligible for inclusion if they met the following criteria:

- They included statements which described the knowledge a person should have, actions a person should take or information or content that should be included when communicating about suicide; AND
- The statement was used in the context of communicating online, or through social media or social networking platforms

Sources from the peer reviewed and grey literature were excluded if they involved actions that are mandated by law (e.g. legislative prohibition against reporting suicide); the article referred to provisions for the delivery of online therapy by a qualified counsellor, therapist or health professional; or the article did not involve communication online or using social media.

### Development of the questionnaire

A total of 73 peer reviewed and grey literature informed the development of the Round 1 questionnaire (N = 42 and N = 31 from the peer-reviewed and grey literature, respectively). Statements were extracted from the relevant literature and reviewed by a working group that comprised four members (NH, PT, JR, and NR) who were experienced researchers in suicide prevention or the Delphi expert consensus method. The working group omitted statements that contained duplicate information, and when required, systematically reworded statements for consistency across multiple online communication platforms. For example, the statement *“When choosing imagery for your blog post*, *avoid selecting clichéd*, *emotional images or graphic depictions of violence or self-harm”* was reworded as *“If posting or sharing an image or video avoid images of people looking dishevelled or threatening*, *or clutching their head*.*”* The final questionnaire consisted of 284 items and was organised into nine topic areas, summarised in Table 1. Two paid youth advisors (ZT and RB) from Orygen were appointed to provide ongoing feedback on all materials developed throughout the duration of the project. All items included in the questionnaire are available in the S1 Table.

**Table 1:** Sections included in the Delphi questionnaire and example items.

Section	Topic	Examples of questionnaire items
1	General tips	Don’t say “committed suicide” Do say “died by suicide” Do not use clichéd, emotional images (e.g., a person holding their head in hands)
2	Things to consider before you post online	Be aware that what goes online may be there forever Be aware that once your post is made public you have no control over who will see it, or who will share it
3	Communication about someone you know who is affected by suicide	Do not speculate in your post about why the person took their life; Include any efforts that the person made to reduce their suffering;
4	Celebrity suicide	Do not post or share content that speculates the suicide of a celebrity before it has been confirmed by an official source (e.g., a well-known and reliable news website)
5	Writing about your own experience	Consider that others in your life who don’t know about your experience, (e.g. employers or family members), might find out as a result of online disclosure Highlight those parts of your story that support recovery and hope, and have the potential to reduce
6	Monitoring online content	Do check responses to your post regularly for unsafe content in the following circumstances: Your post refers to suicide or suicidal behaviour; You have replied to a post that involves suicide-related content;
7	Responding to someone who may be suicidal	Ask the person at risk directly if they are thinking of suicide (e.g., “are you thinking suicide?”, “are you suicidal?”, “are you thinking of ending your life?”)
8	Responding to harmful comments	Avoid arguing in the comments section; Advise the user that their post is unsafe; Delete the users post if the platform allows it
9	Memorial pages and closed groups to honour the deceased	Include a ‘terms of use’ Ask all potential members/followers of the account, group, or page to read and accept the ‘terms of use’ before approving their membership

### Expert panel formation

Two panels, one consisting of international suicide prevention researchers and media and communications specialists and one consisting of Australian youth advocates (referred to hereafter as the professional panel and youth panel, respectively) were recruited for participation in the study. All panel members were at least 18 years of age and fluent in English. Recruitment and eligibility of panel members are described below.

#### Professional panel

Professional panel members were identified through two primary channels. First, lead authors of articles retrieved via the literature search, and deemed eligible for inclusion, were invited to participate. Second, media and communications specialists were identified via the investigators’ professional networks and included both suicide prevention researchers and media and communications specialists from organisations including Suicide Prevention Australia, Everymind, and Lifeline Australia. Professional panel members were eligible to participate in the study if they were: 1) At least 18 years of age; AND 2) Employed as a media or communications professional OR an academic or researcher in suicide prevention.

#### Youth panel

Youth panel members were recruited via Orygen’s youth advisory network. Orygen is Australia’s National Centre of Excellence in Youth Mental Health. Orygen is located in Melbourne, Australia and conducts world-leading research into the full spectrum of youth mental health difficulties. It also houses an integrated clinical service, an education and training department and a policy and advocacy unit. The purpose of the youth advisory network is to provide a platform for young people to advocate for youth mental health and an opportunity for them to be involved in youth mental health research. Youth panel members were eligible to participate in the study if they were: 1) Aged between 18 and 25 years; AND 2) Resident in Australia; AND 3) A current member of Orygen’s youth advisory network.

A minimum of 20 members is necessary for each panel in order to prevent individual panel members from having a disproportional effect in Delphi expert consensus studies [24]. As noted above, the total number of panel members in the current study was 70 (43 professionals and 27 youth). The proportion of panel members that identified as female was 73.3% (65.1% professionals and 81.5% youth). The mean age was 43.9 years for professionals and 22.4 years for youth. Of the professional panel members, 15 (34.9%) were media professionals or communication specialists, and 28 (65.1%) were suicide prevention researchers, some of whom were also employed in a clinical role. The professional panel consisted of a broad panel of international experts including members from Australia, Austria, Canada, Estonia, Hong Kong, Ireland, New Zealand, South Korea, Switzerland, the United Kingdom and the United States. All panellists were invited to participate via an email sent by the lead investigator (JR).

### Delphi consensus ratings

Panel members were asked to complete two rounds of questionnaires distributed online using the web-based survey software ‘Qualtrics’. Panel members were advised that their participation in the Delphi study would lead to the development of a set of guidelines for young people involving safe communication about suicide online. During both the Round 1 and Round 2 questionnaires panel members were instructed to rate each item according to its importance for inclusion in the guidelines using a five-point Likert scale that included the following options: *essential*, *important*, *unsure/depends*, *unimportant*, *should not be included*.

#### Round 1 questionnaire

During the Round 1 questionnaire, panel members were presented with the original 284 item questionnaire outlined above. Panel members were given the opportunity to provide feedback or suggest additional strategies in free text at the end of each section. The working group reviewed all feedback from panel members and any new items that represented an original idea, not otherwise included in the Round 1 questionnaire was included as a new item in the Round 2 questionnaire. As in previous studies [25–27], items were eligible for inclusion in the guidelines if 80% or more of both the professional and youth panels endorsed an item as essential or important. Items were re-rated in the Round 2 questionnaire if 80% or more of one panel endorsed an item as essential or important, or items were rated as either essential or important by 70–79.5% by both panels. Any items that did not meet the above conditions were excluded from the Round 2 questionnaire.

#### Round 2 questionnaire

The Round 2 questionnaire comprised of 85 items (62 re-rate items from the Round 1 questionnaire and 23 new items provided as feedback by panel members in the Round 1 questionnaire). All panel members were provided with a summary report that included a comparison of the panel member’s individual ratings against the overall response of the professional and youth panel, for each item. Panel members were instructed to use this feedback for consideration during the re-rating of items in the Round 2 questionnaire [22]. Panel members were instructed to rate the items as outlined above. The criteria for consensus in the Round 2 questionnaire included all items endorsed as essential or important by at least 80% or more of both panels. All other items in the Round 2 questionnaire, which did not meet the criteria for consensus by at least 80% of both panels, were considered not eligible for inclusion in the guidelines.

### Development of the #chatsafe guidelines

The authors from the working group combined all items (i.e. those that received >80% consensus for inclusion in the guidelines) that contained similar content, and combined them into prose, and wrote them into the actual guidelines. The working group and two project youth advisors (ZT and RB) reviewed the guidelines and ensured that the content and language was youth and social media friendly, and where relevant, provided some practical examples to be incorporated into the guidelines. Careful consideration was given to ensuring that the final guidelines were true to the original wording of the questionnaire items whilst still being coherent and easy to read. The draft guidelines were then provided to panel members for final feedback and endorsement. No changes to the content of the guidelines were made at this stage. A copy of the #chatsafe guidelines are available in the S1 File.

## Results

**Fig 1:**
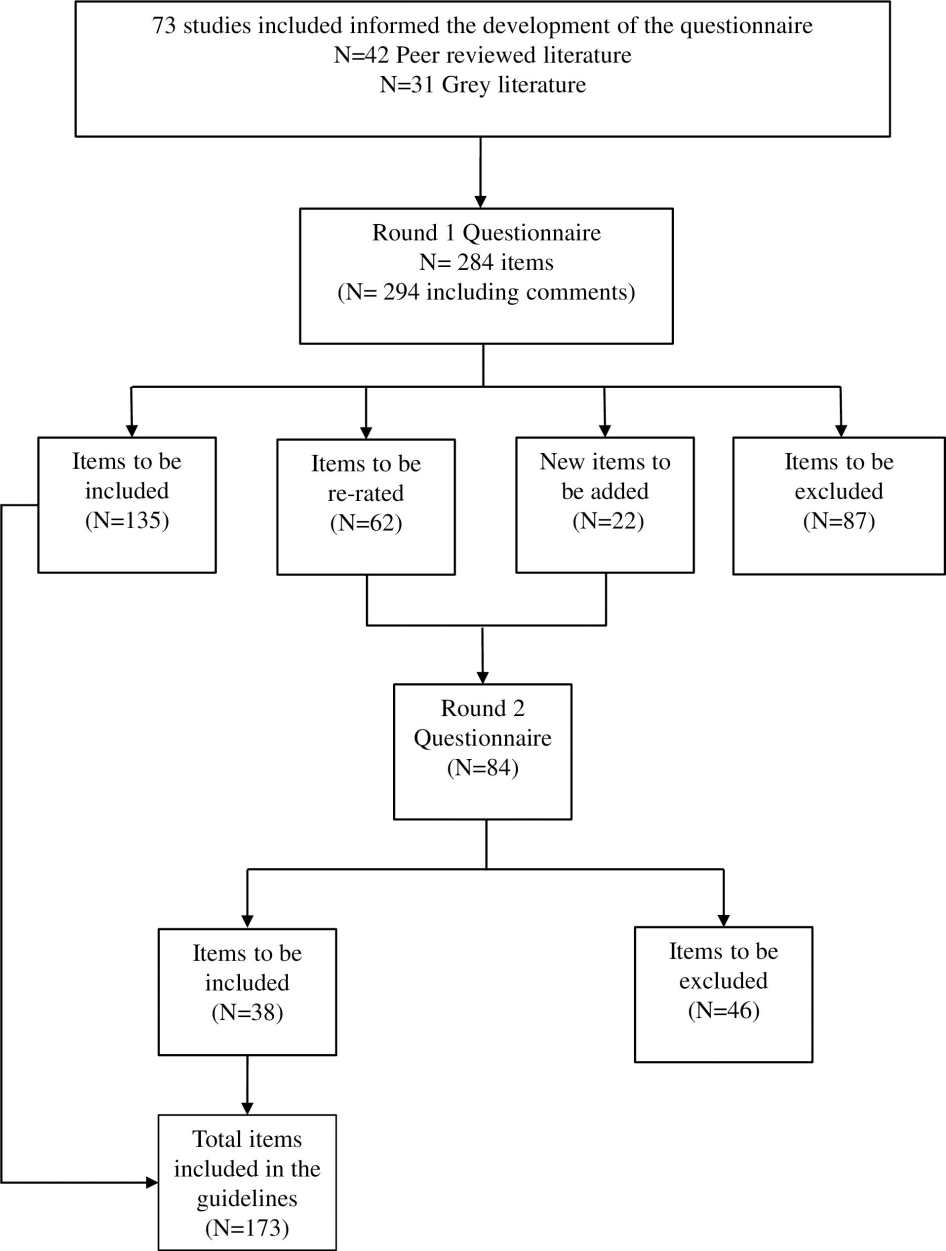
Flow chart of included studies and questionnaire items by Delphi round.

Participation rates of the professional and youth panels are shown in Table 2. Overall, the participation rate of panel members who completed both questionnaire rounds was 84.3% (83.7% professionals and 85.2% youth). The total number of items included, excluded and re-rated in each round is shown in Fig 1. Both panels rated a total of 305 individual items (284 items from the original Round 1questionnaire, and 22 new items based on feedback from panel members during Round 1). Three duplicate items that received similar consensus were excluded from the Round 2 questionnaire. Overall, 171 out of 305 items (165 original questionnaire items and 6 feedback items from panel members) were rated as ‘essential or ‘important’ by at least 80% of both panels and were thus were included in the guidelines (See S1 Table for individual item results in Round 1 and Round 2)

**Table 2:** Participation rates of panel members.

	Round 1 (N)	Round 2 (N)	Completion (%)
**Youth**	27	23	85.2%
**Professional**	43	36	83.7%
**Total**	**70**	**59**	**84.3%**

There was strong agreement between items rated as ‘*essential’* or ‘*important’* by both the professional and youth panels (r = 0.86, p < .001). Panel members were asked to rate items that covered the appropriate language to use when communicating about suicide, and endorsed items including avoiding language that glorifies suicide, and avoiding terms that connote suicide as criminal e.g., the term ‘*commit suicide’*. Despite this, there were some areas in which panel member ratings differed. For example, items, which received notably higher ratings from the professional panel included taking greater care in the use of stigmatising language and in the use of images or video content that depicts methods of suicide. By contrast the youth panel were more likely to endorse statements involving: communication about suicide in general; strategies to help them safely share their own suicidal thoughts, feelings and behaviours; and statements relating to monitoring their posts, and reaching out to someone who may be suicidal. Youth panel members were also more likely to endorse statements relating to the use of content or trigger warnings and those relating to the terms of use in closed groups or memorial pages.

The final guidelines are available as a supplementary file (see S1 File). They were organised into five sections that followed the structure of the questionnaire and reflected the ways in which our youth networks reported using social media for the purpose of communicating about suicide. These sections include the following 1) Before you post anything online about suicide; 2) Sharing your own thoughts, feelings or experience with suicidal behaviour online; 3) Communicating about someone you know who is affected by suicidal thoughts, feelings or behaviours; 4) Responding to someone who may be suicidal; 5) Memorial websites, pages and closed groups to honour the deceased.

## Discussion

This study employed the Delphi expert consensus method to develop a set of evidence-informed, and publicly available guidelines to help young people communicate safely about suicide via social media. To the best of our knowledge it is the first study internationally to develop guidelines that are specifically designed for young people and for social media.

### How the #chatsafe guidelines compare to traditional media guidelines

As noted above, guidelines to assist professionals report safely about suicide have existed for many years and form a key component of several suicide prevention strategies around the world [28–30]. The guidelines developed in the current study have some similarities to extant guidelines, but they also include advice relating to some of the issues specific to the amorphous and interactive nature of social media. For example, Section One ‘*Before you post anything online about suicide*’ details the type of content and language that is generally considered to be unhelpful when communicating about suicide, such as graphic descriptions or images of suicide methods or locations. It also includes examples of types of language that should be avoided, such as terms like ‘*committed suicide’* which portrays suicide as a criminal act and as such may be stigmatising for some, or terms like ‘*successful suicide attempt’*, which may hold a positive connotation. There has been significant debate in recent times about appropriate language to use when communicating about suicide [31, 32]. To date, inconsistencies in the nomenclature of suicidal behaviours, has not only limited comparison between studies, but also, at times, may be perceived as pejorative towards individuals who have experienced suicidal behaviours, and their friends and relatives [33, 34]. In the current study the two panels rated a number of language-related items and endorsed the term ‘*died by suicide’*. They also endorsed items advising that the complexity of suicide, and messages of hope and recovery be conveyed in posts about suicide. This reflects research that has shown stories that portray an individual gaining a sense of mastery over their problems may have a preventative effect [11]. For example, in a study of media reports of suicide in Austria, Niederkrotenthaler and colleagues (2010) demonstrated that newspaper reports that included stories of suicide ideation, not accompanied by a suicide attempt or suicide death was associated with a decrease in suicide rates, and thus may have a protective effect. However until now this has only been included in media guidelines targeting journalists and has not been used to help educate or inform young people.

The current guidelines also provide young people with advice on how to respond to someone who has indicated suicide risk. Previous studies have shown that young people are more likely to engage in social media content that depicts suicidal behaviour [35] and endorse taking action to assist those who share suicidal content online [36]. Nevertheless, concerns have previously been expressed about the safety of talking about suicide on social media for the reasons cited above. However, rather than advising young people against communicating in this way, the guidelines include specific tips on how to have these conversations safely and provide some examples of language to use if they are worried about someone who is suicidal (e.g., ‘*Are you thinking of suicide*?’ or ‘*Do you feel suicidal*?’) They then give young people a series of options on how to respond. Importantly, the #chatsafe guidelines inform young people that it is ok not to respond to someone who may be suicidal. The notion of self-care and personal wellbeing are emphasised throughout the guidelines, and include additional strategies such as taking a break from social media, taking control of content which may cause distress, and reflecting on one’s emotional state and ability to respond to suicide-related content. These recommendations are particularly important given the maturation of complex cognitive, and socio-emotional processes that influence decision making and affective regulation in young people are in a state of ongoing development [37].

Unlike other guidelines, the #chatsafe guidelines also advise on issues specific to the online environment. For example, Section 1 includes a number of items that remind young people about issues relating to privacy, to the permanency of content posted online, to the speed at which content can spread, and the lack of control one might have over content once it is posted [38, 39]. Additionally, the final section of the guidelines focuses on how to operate closed groups and memorial pages safely. These groups and pages are often established following a suicide death by those who wish to memorialise their friend or loved one; they can also be supportive environments that provide a sense of community [40]. However, concerns have been expressed about how to operate these pages safely, and regarding their possible impact on suicide contagion and clusters [41, 42], hence reiterating the need for evidence-informed guidance in this area.

### Involving young people in the study

The involvement of young people was central to this study. To the best of our knowledge only very few Delphi expert consensus studies that focus on mental-health related topics have included young people as panellists [26, 43]. This may be explained by the reluctance to involve young people in certain types of research, and in particular suicide-related research, for fear of iatrogenic effects [44]. However, there is an emerging body of evidence to suggest that it is indeed safe and acceptable to include young people in suicide prevention research, including online research [45], and given that the #chatsafe guidelines are designed for young people, their active participation was crucial.

Of interest are some of the items that did not achieve consensus due to the differing views of young people and professionals. One example is the item ‘*If you see a post and are worried that someone may be suicidal always reach out to them directly*’. This item was endorsed by the youth panel as important to include but not by the professional panel and was therefore excluded from the guidelines. This lack of consensus may reflect the different ways in which the younger and older generations engage with social media [46]. Young peoples’ on- and off-line worlds are far more integrated than those of the older generations [47], with the youth panel making comments such as ‘*of course we would reach out directly to someone we were worried about’*. By contrast, the professionals raised concerns about issues such as the complexity of responding to someone at risk of suicide and young people assuming too much responsibility.

### Implementing the guidelines

Critical to uptake of guidelines is the way in which they are implemented, and in this case the #chatsafe guidelines will be implemented via a large-scale social media campaign. The delivery of population-wide media campaigns has gained attention recently as a potentially effective suicide prevention strategy, with the evidence suggesting that they have the capacity to increase knowledge and awareness of suicide, and improve attitudes towards help-seeking [48]. To date, however, there is limited evidence regarding the impact of such campaigns on young people, and few, if any, actively involve young people in their design and implementation. We recently conducted a small pilot study with secondary school students that examined the feasibility and safety of co-developing suicide prevention campaign materials for delivery via social media. Young people responded positively to the project, reporting it to be enjoyable and educational and no adverse events were reported [49]. Thus, building on the findings from the previous pilot, the #chatsafe guidelines developed in the current study will now be brought to life via the development of a co-designed social media campaign developed by and for young people.

## Conclusions

This is the first study in any country to develop a set of evidence-informed guidelines designed to support young people to talk safely about suicide on social media. Whilst designed for an Australian audience in mind they can readily be adapted for other settings. In Australia the guidelines will be implemented via a co-designed suicide prevention social media campaign. It is hoped that the guidelines and campaign materials will not only be a useful resource for young people themselves, but will also be used by those who support young people, including parents, teachers, community workers and health professionals.

## Supporting information

S1 Table Delphi Expert Consensus item results for Round 1 and Round 2. (PDF)

S1 File #Chatsafe A young person’s guide for communicating safely online about suicide. The authors have received permission from the copyright owner of this file to publish it under a CC BY 4.0 license.
(PDF)
