# Supplementary material for: The #chatsafe project. Developing guidelines to help young people communicate safely about suicide on social media: A Delphi study
Source: PLoS One. 2018 Nov 15;13(11):e0206584. doi: 10.1371/journal.pone.0206584 (PMC6237326; doi:10.1371/journal.pone.0206584)
Supplement: S1 Table — (PDF) [file pone.0206584.s001.pdf]

S1 Table. Delphi Expert Consensus item results for Round 1 and Round 2

		Percent rated as essential or important					
		Round 1			Round 2		
**Item #**	**Statement**	Youth	Professional	Item result	Youth	Professional	Item result
**Section 1. General tips**							
	**DO use the following phrases when talking about suicide:**						
1	"Died by suicide"	85.18%	90.70%	Include*			
2	"Killed themselves"	7.41%	46.51%	Exclude			
3	"Took their own life"	66.67%	72.10%	Exclude			
4	"Attempted suicide"	74.08%	76.74%	Rerate	73.91%	63.89%	Exclude
5	"Completed suicide"	40.74%	25.58%	Exclude			
6	"Survived a suicide attempt"	77.78%	72.09%	Rerate	73.91%	55.56%	Exclude
7	"Made a suicide attempt"			New item*	69.56%	63.89%	Exclude
8	Do not say "committed suicide"	88.89%	83.72%	Include*			
9	Do not normalise suicide ideation			New item	82.61%	63.89%	Exclude
10	Don't say suicide is common, normal, or usual for any person,	55.55%	86.05%	Rerate	69.57%	72.22%	Exclude
11	Don't say that suicide is a solution to a problem, life stressors,			New item	91.31%	88.89%	Include
12	Don't suggest suicide is an understandable, or acceptable	62.96%	74.42%	Exclude			
13	Emphasise that suicide is not a normal reaction to mental	59.26%	65.11%	Exclude			
14	Tell others who might be contemplating suicide how they could get help	100.00%	97.68%	Include			
15	Indicate suicide is preventable, help is available, treatment can be successful, and that recovery is possible	100.00%	95.35%	Include			
16	Don't describe suicide as an "epidemic"; "outbreak"; "at an all time high"; "alarming rate"; "skyrocketing"; or "increasing	74.08%	86.04%	Rerate	73.91%	88.89%	Exclude
17	Don't exaggerate, or use provocative adjectives, such as "deadly addiction"; "disastrous relationship breakdown";	77.77%	86.05%	Rerate	78.26%	91.67%	Exclude
18	Don't provide links to sensational "clickbait"	85.18%	95.35%	Include			
	**DO use the following nouns and phrases when posting about**						
19	"Increase"	81.49%	67.45%	Rerate	86.96%	52.78%	Exclude
20	"Rise"	70.37%	60.47%	Exclude			
21	"High rates"	77.78%	55.81%	Exclude			
22	"Increasing rates"	81.48%	58.14%	Rerate	69.57%	55.56%	Exclude
23	"Clusters of death"	3.70%	32.56%	Exclude			
24	"Concerning rate"	74.08%	53.49%	Exclude			
25	Don't 'share' the content of a suicide note or message	70.37%	83.72%	Rerate	95.66%	88.89%	Include
26	Don't quote the content of a suicide note or message			New item	82.61%	86.11%	Include
27	Don't 'like' suicide notes or messages shared online	81.48%	88.37%	Include			
28	Don't only focus on the deceased person's positive	44.45%	37.21%	Exclude			
29	Don't 'like' posts that involve a person who has died by suicide	14.81%	37.21%	Exclude			
30	Don't post about or include links to pro suicide sites or forums	92.59%	97.67%	Include			
31	Don't post about, or share, information about suicide pacts	92.60%	90.70%	Include			
32	Say the person died by suicide, and leave it at that	55.55%	72.09%	Exclude			
33	Say the person left a suicide note or message, and leave it at	18.52%	41.86%	Exclude			
34	Indicate the consequences of the person's death on family,	62.97%	62.79%	Exclude			
35	Don't use words such as"successful," "unsuccessful,", or "failed attempt"	92.60%	95.34%	Include			
36	Don't mention any positive consequences of suicidal behaviour	85.19%	88.37%	Include			
37	Don't say death is a "relief", or that the person is "finally at	74.07%	81.39%	Rerate	100.00%	86.11%	Include
38	Don't say that suicide is "quick" or "painless"	77.78%	88.37%	Rerate	100.00%	97.23%	Include
39	Mention the potential physical consequences of a suicide attempt (e.g., pain and permanent disability)	66.67%	65.11%	Exclude			

40	Don't say a suicide was "achieved" , was a "noble end" , or that it was a "brave act"	88.89%	93.03%	Include			
41	Include information on the person's suffering and the lasting impact it has on family, friends, and the wider community	77.78%	69.77%	Rerate	78.26%	58.33%	Exclude
42	Indicate that most individuals who die by suicide struggled with			New item	73.91%	58.33%	Exclude
43	Provide positive details about the person's life and their			New item	91.31%	66.67%	Exclude
44	Provide information on how the person contributed to society			New item	86.95%	52.78%	Exclude
45	Acknowledge the positive characteristics and achievements of the person's life, but separate these from your post on the			New item	86.95%	66.66%	Exclude
46	Don't blame one event, or imply the suicide was the result of a single cause, such as bullying or social media	96.29%	93.02%	Include			
47	Indicate that suicide is complex and many factors contribute to a person ending their life	96.30%	95.35%	Include			
48	Indicate that most individuals who die by suicide struggled with	77.78%	65.12%	Exclude			
49	Don't say suicide is for "cowards", a "cry for help", or "attention seeking"	85.18%	93.02%	Include			
50	Don't describe suicide as a "selfish" act	81.48%	88.37%	Include			
51	Don't use stigmatising words, such as " crazy", "psycho", "nuts", "lunatic", "deranged", "defective", "insane", or "abnormal"	88.89%	100.00%	Include			
52	Don't suggest nothing can be done about suicide	88.89%	93.03%	Include			
53	Don't suggest that if someone wants to end their life then there is nothing you could do	88.89%	95.35%	Include			
54	Include messages of hope and recovery	96.30%	95.35%	Include			
55	Include links to sources of help (e.g., Kids Helpline, and eheadspace)	96.29%	100.00%	Include			
56	Include links to websites which contain information about suicide prevention	100.00%	95.35%	Include			
57	Inform others that suicide could be prevented	96.30%	90.70%	Include			
58	Include information on factors that protect against suicide (e.g., building and maintaining meaningful connections and relationships)	100.00%	90.69%	Include			
59	Don't provide information about suicide methods (i.e., how the person died)	88.89%	93.03%	Include			
60	Don't provide information about the location of suicide (i.e.,	74.07%	88.37%	Rerate	86.96%	83.33%	Include
61	Don't refer to the fact that there have been a number of suicide acts at a specific location	81.48%	83.72%	Include			
62	Don't label locations as suicide hotspots	81.48%	86.04%	Include			
	**DO NOT use the following if you are posting, adding, or sharing photos, images, or video content:**						
63	Clichéd, emotional images (e.g., a person holding their head	44.44%	67.44%	Exclude			
64	Portrait images of those who died by or attempted suicide	29.63%	55.82%	Exclude			
65	Images of grieving relatives, friends, peers, or other	40.74%	53.49%	Exclude			
66	Images from the funeral or memorial service	40.74%	60.46%	Exclude			
67	Images of people looking dishevelled, threatening, or	66.66%	76.75%	Exclude			
68	Images or video content that depict method of suicide	85.19%	95.35%	Include			
69	Images or video content that depict method of self-harm	88.89%	95.35%	Include			
70	Images or video content that depict the location of suicide	85.18%	83.72%	Include			
71	Any suicide-related animation	74.07%	79.07%	Rerate	86.96%	80.56%	Include
72	Images of people looking dishevelled, threatening, or			New item	82.61%	86.11%	Include
73	Make sure the face is blurred in portrait images	29.62%	27.91%	Exclude			
74	Choose images that show the individual who died by suicide	62.97%	55.81%	Exclude			
75	**DO provide a trigger warning in your post when your post**						
75	Images of the person who died by suicide	70.37%	51.16%	Exclude			
76	Graphic or descriptive content	100.00%	81.39%	Include			
77	Content that might be distressing for others	100.00%	81.40%	Include			

78	Position trigger warning at the beginning of your post, so readers can make an informed decision that best suits their wellbeing	100.00%	81.40%	Include			
79	Position the trigger warning after a neutral description of your	33.33%	44.18%	Exclude			
80	Include a hyperlink or information for a suicide prevention helpline (this should follow the recommendations in the section on promoting help seeking)	100.00%	88.37%	Include			
81	Use a bold font or a different colour to distinguish the trigger	92.59%	62.79%	Rerate	95.65%	63.89%	Exclude
82	Don't use exclamation marks in your trigger warning			New item	52.18%	52.78%	Exclude
83	Include a checklist of questions or items that determine if the users are 'fit' to see the content in the post			New item	65.21%	33.34%	Exclude
84	Set up your trigger warning with a few links, or dashes in between, so that people have to click, or scroll before viewing			New item	100.00%	61.11%	Exclude
**Section 2. Things to consider before you post online**							
	**DO the following before you post anything about suicide**						
85	Be aware that it's OK to post about suicide	88.89%	65.12%	Rerate	95.66%	66.66%	Exclude
86	Take some time to think about why you want to share this post on social media	100.00%	97.67%	Include			
87	Be aware that what goes online may be there forever	100.00%	95.35%	Include			
88	Be aware that once your post is made public you have no control over who will see it, or who will share it	96.30%	93.02%	Include			
89	Be aware that even if you post something privately, it could become public	96.30%	95.35%	Include			
90	Think about how your post could affect others and whether there is a different way to share your information	100.00%	97.68%	Include			
91	Be aware that everyone is entitled to privacy, even if they die by suicide	96.29%	86.04%	Include			
92	Never share other people's personal information without their permission	100.00%	93.03%	Include			
93	Be aware of some of the warning signs of suicide risk	100.00%	93.03%	Include			
94	Be aware of the suicide prevention resources each platform offers (e.g., Facebook Suicide Prevention)	96.30%	88.37%	Include			
95	Be aware that inaccurate, stigmatising, or unsafe information can quickly go viral'	100.00%	95.35%	Include			
96	Be aware that sometimes repeated exposure to negative content can make you feel bad. It's also possible that a one-off post may trigger negative thoughts and feelings	96.29%	88.37%	Include			
97	Take control of the content you see. For example, if you are going through a rough patch, you may want to minimise the amount of suicidal posts you see	100.00%	93.02%	Include			
98	If things are getting too much, take a break, and talk to someone about how you're feeling	96.30%	97.68%	Include			
99	Have a plan if you feel distress, or are troubled by negative thoughts and feelings due to posts you have shard or comments you have/will see on social media	100.00%	93.03%	Include			
**Section 3. Communication about someone you know who is affected by suicide**							
	**DO NOT do the following when communicating about someone you know who has died by suicide:**						
100	Speculate in your post about why the person took their life	92.59%	95.35%	Include			
101	Speculate about an individual's thoughts and feelings leading up to the suicide	88.89%	90.70%	Include			
102	Post or share inaccurate information	100.00%	100.00%	Include			
103	Share information about the person that you are unsure of	96.29%	100.00%	Include			
104	Mention the person's name or other characteristics that	66.66%	55.81%	Exclude			
105	Explain suicide news in ways of feng shui or metaphysics	77.77%	53.49%	Exclude			
106	Promote memorial sites for the deceased	44.45%	44.18%	Exclude			
107	Only post what you know to be true	88.89%	81.40%	Include			

108 Encourage others not to post or share information that hasn't been confirmed	92.59%	97.67%	Include			
109 Include details that might help put the suicide into context (e.g., warning signs, previous suicide attempts, or if they had	59.26%	51.17%	Exclude			
110 Refer to the person's suffering prior to their death	48.14%	39.53%	Exclude			
111 Include any efforts that the person made to reduce their	37.04%	46.51%	Exclude			
112 Indicate that most people provided clues that they were	55.56%	44.19%	Exclude			
113 Describe any efforts the person made to reduce their suffering	37.04%	34.88%	Exclude			
**DO NOT do the following when communicating about someone you know who is indicating suicidal ideation**						
114 Mention the person's name, or other characteristics that	85.18%	79.07%	Rerate	91.31%	72.23%	Exclude
115 Post something you would not say directly to the person	96.29%	86.05%	Include			
116 Do Contact the person who the post is about	70.37%	69.77%	Exclude			
117 Do Inform a trusted adult	96.29%	90.70%	Include			
118 Do Seek professional advice	92.59%	81.39%	Include			
119 Do Directly contact the person who has posted the information	77.78%	62.79%	Exclude			
120 Do Ask for the permission of the person before posting or sharing content about them	92.59%	81.40%	Include			
121 Do Talk to the person if you are concerned that they are experiencing suicidal feelings or behaviour, before posting your concerns online	96.30%	88.37%	Include			
122 Do Contact the person who the post is about			New item	82.61%	52.77%	Exclude
123 Do Directly contact the person who has posted the information			New item	91.31%	55.56%	Exclude
**Section 4. Celebrity suicides**						
**DO NOT do the following when communicating about a**						
124 Post or share content that speculates the suicide of a celebrity before it has been confirmed by an official source (e.g., a well-known and reliable news website)	92.60%	90.70%	Include			
125 Post or share too many posts about the celebrity suicide in succession, as too much exposure to negative content can have a suggestive effect on vulnerable people	96.30%	95.35%	Include			
**DO the following if you do want to pay tribute to a celebrity**						
126 Provide positive details about the celebrity's life and their	81.48%	72.09%	Rerate	95.65%	77.78%	Exclude
127 Provide information on how the celebrity contributed to	85.18%	74.42%	Rerate	91.30%	69.45%	Exclude
128 Acknowledge their positive characteristics and achievements, but separate these from your post on the			New item	78.26%	77.78%	Exclude
**DO the following if you do want to pay tribute to a celebrity**						
129 Explain how the celebrity death negatively impacts others	59.26%	53.49%	Exclude			
130 Provide links to helplines	96.30%	95.35%	Include			
**Section 5. Posting about your own experience**						
**DO the following before deciding to post about your**						
131 If you are currently experiencing suicidal thoughts, feelings, or behaviours talk to a trusted adult, friend, or peer, or seek professional help from a healthcare professional, before posting online. If you have a safety plan, use the strategies outlined in it	96.30%	93.02%	Include			
132 Consider that others in your life who don't know about your experience, (e.g. employers or family members), might find out as a result of online disclosure	85.18%	86.04%	Include			
133 Think about which parts of your personal experience you wish to disclose (e.g., you don't have to share everything about your personal story)	100.00%	88.37%	Include			
134 Ask yourself how you think disclosing your personal experience will make you feel?	100.00%	90.70%	Include			

135	Ask yourself what you hope to achieve by sharing this post (e.g., do you want to raise awareness, or are you looking for support?) and are there more effective ways of achieving that?	100.00%	95.35%	Include				
136	Ask yourself if you would you benefit from seeking help, and, what resources, people or friends are available to help you with that?	92.59%	97.68%	Include				
137	Ask yourself who will see your post? For example, are you posting on a professional mental health site, an anonymous platform, a public platform?	92.59%	86.05%	Include				
138	Ask yourself how your post might affect your friends, family and peers? (e.g., will your audience know how to help themselves or their loved ones after reading your story?	88.89%	81.39%	Include				
139	Do display hyperlinks to support services and help-seeking information clearly at the top of the page/begging of the post			New item	100.00%	94.45%		Include
140	Have a plan in place about what to do if a person indicates feeling distressed as a result of your post (e.g., providing	100.00%	79.07%	Rerate	100.00%	94.45%		Include
	**DO the following once you have decided to post about your**							
141	Write out your story in full in a private format	44.45%	51.17%	Exclude				
142	Take out any information you would not want everyone you have ever met, or will ever meet, to know about you	91.67%	76.26%	Rerate	100.00%	97.23%		Include
143	Take out any graphic references to self-harm or suicide	83.33%	86.78%	Include				
144	Highlight those parts of your story that support recovery and hope, and have the potential to reduce stigma	95.84%	76.19%	Rerate	100.00%	100.00%		Include
145	Include physical health information if it relates to a disability sustained as a result of a suicide attempt			New item	43.48%	44.45%		Exclude
146	Remove any identifiable personal information	62.50%	46.38%	Exclude				
147	Remove any physical health information	29.16%	21.99%	Exclude				
148	Remove any mental health information	25.00%	19.36%	Exclude				
149	Only disclose the personal details you feel comfortable letting others know about	100.00%	97.29%	Include				
150	Include the challenges you faced	59.26%	62.79%	Exclude				
151	Include things and people that supported your recovery	92.60%	88.37%	Include				
152	Include your personal experience seeking help	85.18%	76.74%	Rerate	78.26%	75.00%		Exclude
153	Include positive experiences you had when seeking help	96.29%	90.69%	Include				
154	Include thoughts and feelings you experienced that others	88.89%	65.12%	Rerate	82.61%	66.66%		Exclude
155	Include things that facilitated your recovery and how you coped	96.29%	83.72%	Include				
156	Highlight the importance of seeking help early	92.60%	86.04%	Include				
**Section 6. Monitoring online content**								
157	Do Monitor online content	92.59%	69.77%	Rerate	95.66%	80.55%		Include
	**DO check responses to your post regularly for unsafe content**							
158	Your post refers to suicide or suicidal behaviour	96.30%	92.50%	Include				
159	Ask a trusted person to monitor online content			New item	86.96%	72.22%		Exclude
160	You have replied to a post that involves suicide-related	85.19%	72.50%	Rerate	91.31%	86.11%		Include
161	You have seen a post that involves suicide-related content regardless of whether you are the original poster or have	77.78%	56.41%	Exclude				
	**DO set up your phone with instant notifications (e.g., badges,**							
162	You are monitoring your own post	70.37%	57.50%	Exclude				
163	You are monitoring someone else's post	48.15%	40.00%	Exclude				
164	Whenever you have seen a post that involves suicide	40.74%	22.50%	Exclude				
	**DO the following if you allow comments, but choose not to**							
165	Disable the comments and feedback option	40.74%	48.83%	Exclude				
166	Include an explicit statement that indicates responses to	59.26%	74.42%	Exclude				

167	Provide the phone numbers or hyperlinks to appropriate helplines, suicide prevention services, or emergency services for your local area (e.g., in Australia, you might include services, such as Lifeline, Kids Helpline, e-headspace)	92.59%	93.02%	Include			
**Section 7. Responding to someone who may be suicidal**							
	**DO ask yourself the following before responding to someone**						
168	How are you feeling?	92.59%	81.39%	Include			
169	Are you able to provide support to the person, if needed?	100.00%	95.34%	Include			
170	Is there someone else who could help?	96.29%	97.67%	Include			
171	Will responding to the person make you feel unsafe or distressed?	100.00%	90.69%	Include			
172	Are you confident about how to deal with this situation?	92.59%	90.70%	Include			
173	Do you understand your role and the limits of the support you could provide?	100.00%	93.03%	Include			
174	Do you know where you could signpost this person to for more support?	100.00%	95.35%	Include			
	**DO the following if you see a post and think someone may be**						
175	Set boundaries about the type of support you are able to offer and when	92.59%	83.72%	Include			
176	Do not place yourself in physical danger	96.30%	93.03%	Include			
177	Put your own wellbeing first and think about whether you could ask for someone else to help	100.00%	88.38%	Include			
178	Report the post to the appropriate social media Safety Team or Help Centre (e.g. Facebook suicide prevention help	77.78%	74.42%	Rerate	91.31%	88.88%	Include
179	Always reach out to the person at risk directly, even if you think that they may not intend to follow through	81.48%	58.14%	Rerate	95.65%	66.67%	Exclude
180	Always respond to messages that indicate suicide risk in private (e.g., through DM or PM), as the conversation may	85.19%	74.42%	Rerate	100.00%	83.33%	Include
181	Always respond to messages that indicate suicide risk both	33.33%	39.54%	Exclude			
182	If you can't contact someone privately, reply once to their	59.26%	53.49%	Exclude			
183	If the comment is on your own post consider removing it, and contacting the at risk person privately with support	88.89%	69.76%	Rerate	82.60%	80.56%	Include
184	Ask the person at risk to follow-up with you	66.67%	46.52%	Exclude			
185	Follow-up with the person at risk to check they are safe and can remain safe	92.59%	86.05%	Include			
	**DO the following if you respond to the person at risk of**						
186	Ask the person at risk directly if they are thinking of suicide (e.g., "are you thinking of suicide?"; "are you suicidal?"; are	88.88%	76.74%	Rerate	86.96%	91.67%	Include
187	If the person at risk says "yes", ask them if they have a	77.78%	65.11%	Exclude			
188	Look at the person at risk's posts to be able to specify exactly why you are worried about them (e.g., "I just wanted to check in, because you posted X, and I am worried about you. Are you feeling suicidal?")	100.00%	88.37%	Include			
189	Respond to person at risk without judgement, assumptions, or interruptions	100.00%	90.69%	Include			
190	Let the person at risk explain in their own words, do not interrupt, respond without judgement, and let them know you care	100.00%	93.02%	Include			
	**DO the following if you do not respond to the person at risk of**						
191	Contact emergency services	40.74%	44.18%	Exclude			
192	Report suicidal content to platform (e.g., Facebook social	85.19%	79.07%	Rerate	100.00%	91.67%	Include
193	Call a crisis helpline for guidance on how to support the user	77.78%	67.44%	Exclude	78.26%	80.55%	
	**DO the following if the person at risk tells you that they have a suicide plan, or have posted or shared content that indicates**						
194	Encourage the person at risk to call 000	92.59%	74.42%	Include			
195	Encourage the person at risk to go to the emergency	81.48%	72.09%	Rerate	91.31%	72.22%	Exclude

196	Contact emergency services yourself (e.g., 000)	70.37%	58.14%	Exclude			
197	Contact a crisis support service for assistance (e.g., Crisis Assessment and Treatment Team (CAAT))	81.49%	60.46%	Rerate	91.31%	75.00%	Exclude
198	Contact a trusted adult with the person at risk's permission	77.78%	65.12%	Exclude			
199	Contact a trusted adult without the person at risk's	59.26%	62.79%	Exclude			
200	Contact the person at risk's family or someone in their social network who may be able to check on them	77.77%	72.09%	Rerate	91.30%	80.56%	include
201	Refer to the person at risk to a mental health service (e.g.,			New item	95.65%	83.34%	Include
202	Take a screenshot of the relevant post(s)	48.15%	60.46%	Exclude			
	**DO the following if you contact emergency services if the**						
203	Ask for an ambulance if you have the person's full name and	74.08%	48.84%	Exclude			
204	Ask for police if you don't know the person's name and/or	70.37%	62.79%	Exclude			
205	Provide your name and contact details to emergency	92.59%	74.42%	Rerate	91.31%	80.56%	Include
206	Inform emergency services that someone has told you online that they have a plan to end their life	88.89%	88.37%	Include			
207	Relay what the person has said to the emergency services	92.59%	90.70%	Include			
208	Provide details on the date and time of the post to emergency services	92.60%	88.37%	Include			
209	Provide details on the person's identity and location to emergency services, if you know it	96.29%	88.37%	Include			
210	Provide the persons IP address if you know it to emergency services and explain that they can use this to find someone's	66.67%	74.42%	Exclude			
211	Keep the person at risk informed about what is happening	66.66%	60.47%	Exclude			
212	Keep the person at risk informed about what is happening, unless this might increase the risk to them or someone else	77.77%	83.72%	Rerate	100.00%	88.89%	Include
213	Try to maintain a conversation with the person at risk until help arrives	96.30%	88.37%	Include			
	**DO the following if the person indicates they are <u>not</u> at**						
214	Ask "do you want to talk about it?", or "what can I do to help?" Let the person you are concerned about make their own decisions;	100.00%	83.72%	Include			
215	Encourage the person you are concerned about to seek	96.30%	79.07%	Rerate	95.65%	88.89%	Include
216	Ask if the person you are concerned about if they know where to get help?	100.00%	93.02%	Include			
217	Ask the person you are concerned about if they would like the phone number of a crisis support or mental health helpline	92.59%	86.04%	Include			
218	Ask the person you are concerned about if they would like the hyperlink to crisis support or e-mental health service (Kids Helpline chat, or e-headspace)	96.29%	86.04%	Include			
219	Reassure the person you are concerned about that help is available	100.00%	88.38%	Include			
220	Avoid using emoticons/emojis when responding as these could be misinterpreted by the person you're trying to help	40.74%	51.16%	Exclude			
221	Avoid using the like function as this could be misinterpreted by the person you are trying to help	62.96%	69.77%	Exclude			
222	Look at the person's posts to be able to acknowledge their feelings and specify exactly why you are worried about them (e.g., "from what you've posted it seems as though you are [illegible]	96.29%	74.42%	Rerate	95.66%	83.34%	Include
223	Provide the phone number to a crisis support or mental health helpline	88.89%	88.37%	Include			
224	Provide the link to a crisis support or mental health helpline	88.89%	79.07%	Rerate	95.65%	91.67%	Include
225	Provide link to online support services and websites	88.89%	86.05%	Include			
226	Provide information about local support services (e.g.,	96.30%	79.07%	Rerate	100.00%	88.89%	Include
	**DO the following if the person who posts suicide-relatedcontent does not want help, and is not at imminent**						

227	Respect what they tell you. If they say no, do not pressure them. Tell them It's okay if you don't want to talk to me, but it is important that you talk to someone	96.30%	81.39%	Include			
228	If the person doesn’t want to access a particular service, acknowledge their reasons, but encourage the person to try	96.30%	76.74%	Rerate	95.65%	77.78%	Exclude
229	Don't expect a positive response; this person is struggling to cope and may feel angry, upset or ambivalent	96.30%	83.72%	Include			
230	You should not to take this personally “ they may well appreciate your help later when they're feeling better	100.00%	88.37%	Include			
231	If you think you might have said the wrong thing, don't panic; show that you care and that it is understandable that they are going through a tough time	100.00%	88.37%	Include			
232	Tag the post with mental-health services relevant to the	44.45%	37.21%	Exclude			
233	Check in with the person to demonstrate that you care	100.00%	86.05%	Include			
234	Once you have provided all the information and support	48.15%	51.16%	Exclude			
	**DO the following if the person posting suicide-related content is a friend that you know in person:**						
235	Call them and offer help	92.59%	79.07%	Rerate	91.31%	69.44%	Exclude
236	Call them and offer support			New item	95.65%	86.11%	Include
237	Visit them and offer help	81.48%	44.19%	Rerate	60.87%	50.00%	Exclude
238	Visit them if it's safe to do so	96.30%	60.47%	Rerate	82.61%	52.78%	Exclude
239	Contact a mutual friend, so that you can reach out to the	59.26%	65.11%	Exclude			
240	Contact a family member	66.66%	58.14%	Exclude			
241	Don't let embarrassment or concern about offending or upsetting your friend from stopping you from reaching out or offering help	100.00%	88.37%	Include			
**Section 8. Respondng to harmful comments**							
	**DO the following if you come across unsafe comments on**						
242	Avoid arguing in the comments section	92.59%	83.72%	Include			
243	Advise the user that their post is unsafe	81.48%	53.49%	Rerate	73.91%	72.22%	Exclude
244	Delete the users post if the platform allows it	74.07%	55.82%	Exclude			
245	Ask the user to delete their own post and explain why	70.37%	48.84%	Exclude			
246	Hide the users post if the platform allows it	85.19%	65.11%	Rerate	95.65%	88.88%	Include
247	Steer the conversation back to more helpful topics	88.89%	79.07%	Rerate	86.95%	75.00%	Exclude
248	Send a personal message to the person and let them know what kind of feedback or discussion is appropriate and what	74.07%	55.81%	Exclude			
249	Report unsafe content to the relevant social media safety	96.30%	76.75%	Rerate	100.00%	91.67%	Include
250	Block the person from posting further comments	44.44%	30.23%	Exclude			
251	Ask the user of the material to delete the unhelpful post	74.07%	48.84%	Exclude			
252	Ask the user of the original post to hide the unhelpful post	70.37%	41.86%	Exclude			
253	Ask the user of the unsafe post to delete their post	66.67%	41.86%	Exclude			
254	Provide the user with your personal email address or ask	33.33%	11.63%	Exclude			
255	Encourage the person to discuss their issue in a private	70.37%	34.88%	Exclude			
256	If you decide to hide or delete the post, contact the user and explain your reasons for doing so	85.19%	62.79%	Rerate	73.92%	61.11%	Exclude
257	DO NOT Engage with people who 'troll' (i.e, people who deliberately post hurtful or harmful comments)			New item	73.91%	64.70%	Exclude
**Section 9. Memorial pages and closed groups to honour the deceased**							
	**DO the following to ensure the memorial account, group, or**						
258	Make the memorial account, group, or page private, so that only group members who have requested to join the group	59.26%	65.11%	Exclude			
259	Make the memorial account, group, or page public, so that is	0.00%	6.98%	Exclude			
260	Only allow members/followers to comment and interact	66.67%	67.44%	Exclude			
261	Allow everyone to comment and interact with other users	22.22%	4.66%	Exclude			

262	Approve comments before they are publicly displayed	81.48%	67.44%	Rerate	78.26%	80.56%	Exclude
263	Monitor comments for suicide-related or harmful content	100.00%	86.05%	Include			
264	Disable comments and feedback option, if unable to monitor	62.97%	67.45%	Exclude			
	**DO the following if you create a private memorial account,**						
265	Deal with any disagreements or unsupportive comments quickly, as they can discourage people from sharing how	88.89%	76.75%	Rerate	91.31%	83.34%	Include
266	Include terms of use'	88.89%	79.07%	Rerate	91.31%	88.88%	Include
267	Ask all potential members/followers of the account, group, or page to read and accept the terms of usebefore	88.89%	72.10%	Rerate	95.65%	88.89%	Include
268	The terms of useshould be accessible by users via a link at the top of the page	88.89%	72.09%	Include			
	**DO the following if you have a 'terms of use':**						
269	State the purpose of the account, group, or page	100.00%	90.70%	Include			
270	State safety rules for communicating about suicide	100.00%	93.02%	Include			
271	Outline your approval process for comments and feedback from other users	100.00%	83.72%	Include			
272	Explain that violation of the terms will result in having the comment removed	100.00%	88.37%	Include			
273	Outline the steps that will be taken to remove posts, or users, who violate the terms of use	92.59%	86.05%	Include			
274	Explain how you will respond to comments which may indicate that a person is at risk of suicide	96.30%	88.37%	Include			
275	Provide information regarding when an account will be suspended or deleted	92.59%	83.72%	Include			
276	Provide information regarding when and how to report suicidal content to appropriate services hosted by the platform (e.g., Facebook Help Centre)	100.00%	86.04%	Include			
277	Provide a contact form or other method (e.g., email address) for users to report unsafe or concerning content	96.29%	67.44%	Rerate	100.00%	83.33%	Include
278	Encourage users to look after their own wellbeing when engaging with your account, group, or page	100.00%	88.38%	Include			
279	Encourage users to take a break if things are getting too	92.59%	76.75%	Rerate	95.65%	88.89%	Include
280	Encourage users to report unsafe content to the appropriate social media monitoring authority (e.g., Facebook Help Centre)	96.30%	86.05%	Include			
281	Provide a list of common suicide warning signs and triggers	77.77%	72.09%	Rerate	91.30%	77.77%	Exclude
282	Explain when and how to report harmful suicidal content	92.60%	72.09%	Rerate	100.00%	88.89%	Include
	**DO make it clear that you discourage the following in your**						
283	Swearing	81.48%	51.17%	Rerate	47.83%	50.00%	Exclude
284	Racism	100.00%	90.70%	Include			
285	Discrimination of any kind	100.00%	95.35%	Include			
286	Graphic details	96.29%	97.68%	Include			
287	DO Suspend or delete an account if the user is posting or	88.89%	74.42%	Rerate	95.66%	72.23%	Exclude
	**DO the following if you suspend, delete an account, or**						
288	Explain your reasons clearly and carefully to the account user	92.60%	88.37%	Include			
289	Let the account user know you are concerned about their	96.30%	76.75%	Rerate	95.65%	88.89%	Include
290	Encourage the account user to look for more appropriate	88.89%	74.42%	Rerate	95.65%	75.00%	Exclude
291	Try to review suspended accounts regularly	59.26%	44.19%	Exclude			
	**DO the following, in addition to the recommendations outlined in section 7 ' Responding to someone who is at risk of**						
292	Respond to post and comments that indicate suicide risk publicly to show your account, group, or page that you are	44.44%	53.49%	Exclude			
293	Respond to posts and comments that indicate suicide risk	77.78%	48.84%	Exclude			
294	Respond to posts and comments that indicate suicide risk	51.85%	53.49%	Exclude			
	**DO the following if responding to a user using a standard**						

295	Express concern for the person	92.59%	83.73%	Include			
296	Personalise the message by using the person's name	88.89%	83.72%	Include			
297	Reflect the language the person has used to describe their feelings	88.89%	81.40%	Include			
298	Adapt your message to the person's situation (e.g., do not recommend a service if they have indicated they did not find the same service helpful)	100.00%	86.05%	Include			
299	Adapt your message to any barriers they feel are stopping them seeking help (e.g., if they don't have mobile phone credit recommend a service that has a call back feature or online chat)	96.29%	86.05%	Include			
300	Adapt your message to interventions they have tried	81.48%	69.77%	Rerate	86.95%	61.11%	Exclude
301	Minimise repeated use of templates if the person you are responding to has reached out to you before	92.60%	88.37%	Include			
302	Your template should incorporate some help seeking resources such as the hyperlink or contact number of support/helpline services	100.00%	90.70%	Include			

*Duplicate item removed from Round 2 questionnaire
