# Supplementary material for: The #chatsafe project. Developing guidelines to help young people communicate safely about suicide on social media: A Delphi study
Source: PLoS One. 2018 Nov 15;13(11):e0206584. doi: 10.1371/journal.pone.0206584 (PMC6237326; doi:10.1371/journal.pone.0206584)
Supplement: S1 File — The authors have received permission from the copyright owner of this file to publish it under a CC BY 4.0 license. (PDF) [file pone.0206584.s002.pdf]

#chatsafe

A young person's guide for communicating safely online about suicide

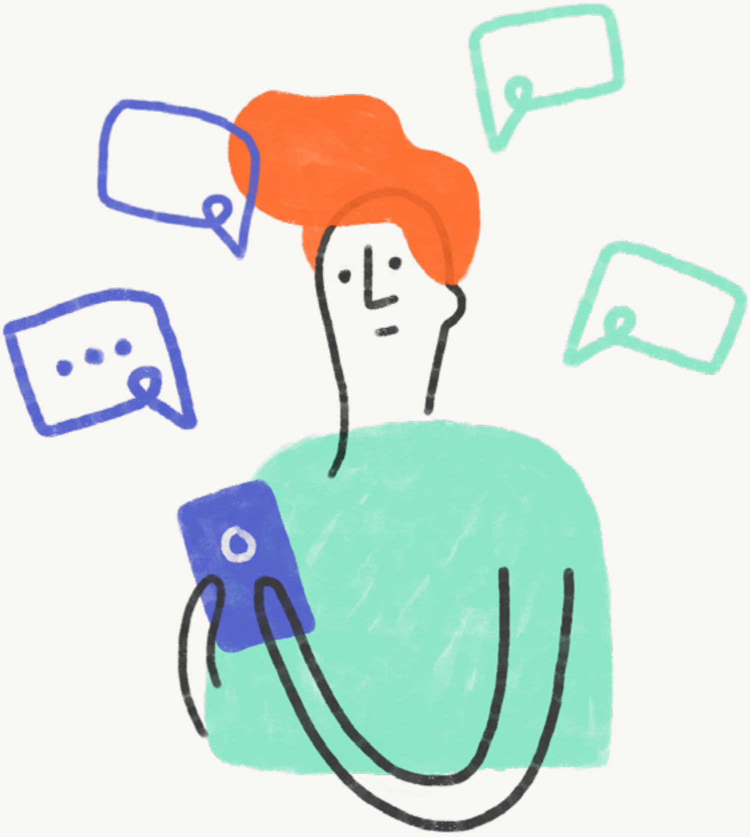

Orygen

The National Centre of Excellence in Youth Mental Health

chatsafe

© Orygen, The National Centre of Excellence in Youth Mental Health

This publication is copyright. Apart from use permitted under the Copyright Act 1968 and subsequent amendments, no part may be reproduced, stored or transmitted by any means without prior written permission of Orygen, The National Centre of Excellence in Youth Mental Health.

ISBN 978-1-920718-72-5

Suggested citation

Robinson, J., Hill, N., Thorn, P., Teh, Z., Battersby, R., & Reavley, N., #chatsafe: A young person's guide for communicating safely online about suicide. Melbourne: Orygen, The National Centre of Excellence in Youth Mental Health, 2018.

Disclaimer

This information is not medical advice. It is generic and does not take into account your personal circumstances, physical wellbeing, mental status or mental requirements. Do not use this information to treat or diagnose your own or another person's medical condition and never ignore medical advice or delay seeking it because of something in this information. Any medical questions should be referred to a qualified healthcare professional. If in doubt, please always seek medical advice.

Orygen, The National Centre of Excellence in Youth Mental Health

Locked Bag 10
Parkville, Vic, 3052
Australia

www.orygen.org.au

Acknowledgements

Orygen, The National Centre of Excellence in Youth Mental Health's #chatsafe Project is supported by funding from the Australian Government, under the National Suicide Prevention Leadership and Support Program.

Orygen, The National Centre of Excellence in Youth Mental Health was first established on the lands of the Wurundjeri people of the Kulin Nation. We pay our respect to Elders past, present and emerging and to Aboriginal and Torres Strait Islander peoples throughout Australia.

The authors thank our project partners: The University of Melbourne, Everymind and Facebook.

The authors thank Simon Goodrich, Ryan Blandon, Libby de Souza and Sarah Kaur from Portable: A digital design and technology company for graphic design and layout.

The authors are extremely grateful to the following expert panel members for their invaluable contribution to the development of these guidelines: Dr John Ackerman, Dr Bart Andrews, Professor Ella Arensman, Eleanor Bailey, Dr Jo Bell, Katherine Berney, Kim Borrowdale, Emily Boubis, Emily Cole, Dr Georgina Cox, Brooke Cross, Han Duong, Bronwen Edwards, Penny Fannin, Marie Gallo-Dyak, Rhylee Hardiman, Amy Hatfield, Professor Keith Hawton, Associate Professor Sarah Hetrick, Taylor Johnstone, Jenya Kalagurgevic, Sharanjit Kaur, Sarah Langley, Tina Li, Britt Liebeck, Dr Ann Luce, Lilian Ma, Nikolina Mabic, Roxxanne MacDonald, Kathryn McGrath, Mary Mansilla, Denise Meine-Graham, Dr Katherine Mok, Dr Sally Morris, Katherine Newton, Associate Professor Thomas Niederkrotenthaler, Meghan O'Keefe, Professor Jong-Ik Park, Emily Parry, Dr Dan Reidenberg, Dr Simon Rice, Ella Robinson-Clarke, Associate Professor Vincent Silenzio, Assistant Professor Mark Sinyor, Professor Merike Sisask, Dr Jaelea Skehan, Ellen Sproule, Andrew Synnot, Assistant Professor Benedikt Till, Dr Michael Utterson, Professor Paul Yip and Assistant Professor Nerissa Young.

We acknowledge young people, as well as their friends and families, who have lived experience of suicide; including those who have attempted suicide and those bereaved by suicide. We remember friends we have lost by suicide and recognise the suffering that suicide brings when it touches our lives and community.

We recognise the communities of young people who are at increased risk of suicide, including Aboriginal and Torres Strait Islander young people, LGBTIQA+ young people, those people in rural and remote areas of Australia and those suffering from mental illness or social disadvantage.

The voices of young people are vital in our suicide prevention work and we join them in hope for the future.

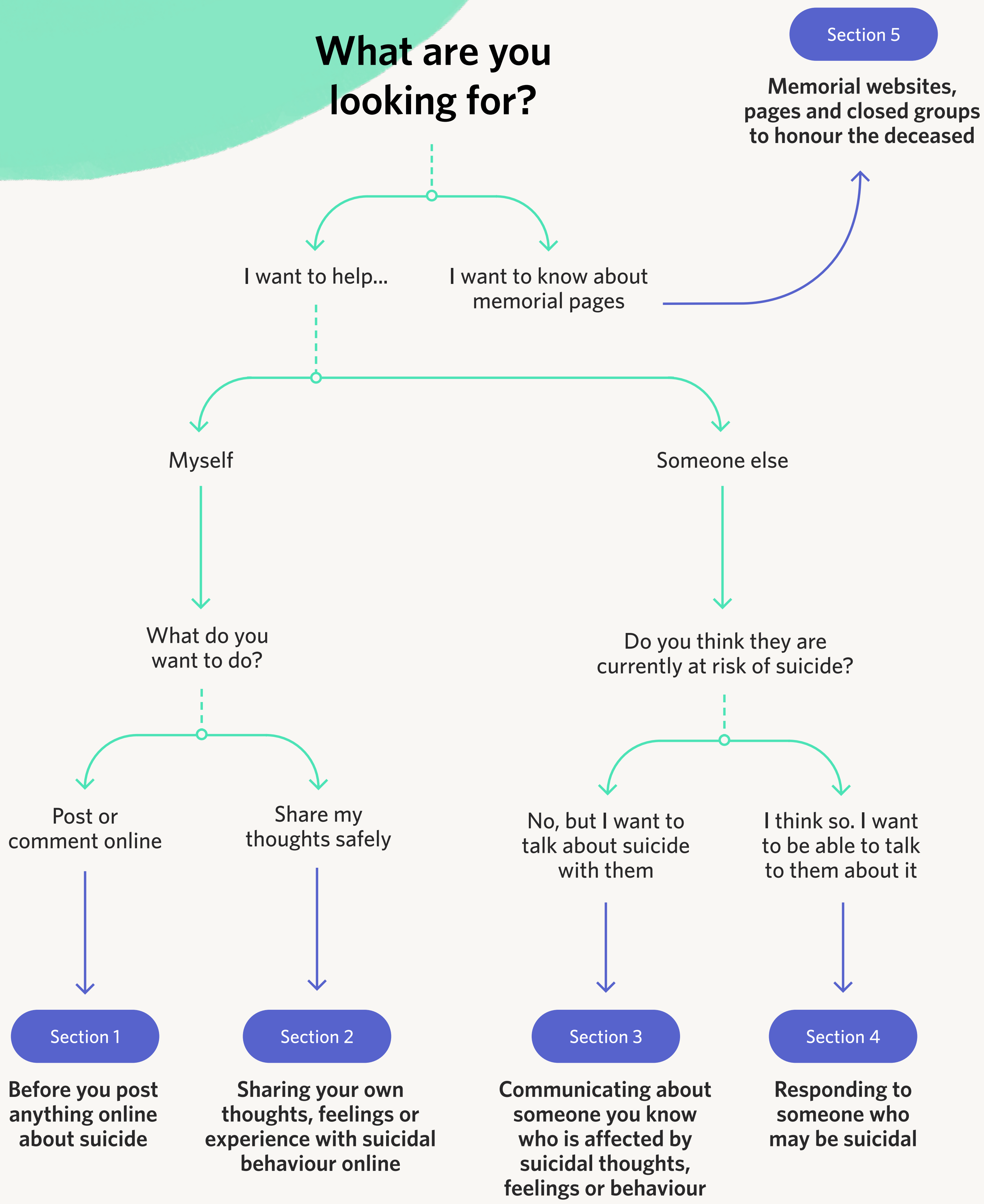
What are you looking for?
I want to help...
I want to know about memorial pages
Section 5
Memorial websites, pages and closed groups to honour the deceased
Myself
Someone else
What do you want to do?
Do you think they are currently at risk of suicide?
Post or comment online
Share my thoughts safely
No, but I want to talk about suicide with them
I think so. I want to be able to talk to them about it
Section 1
Before you post anything online about suicide
Section 2
Sharing your own thoughts, feelings or experience with suicidal behaviour online
Section 3
Communicating about someone you know who is affected by suicidal thoughts, feelings or behaviour
Section 4
Responding to someone who may be suicidal

Background

Many countries, including Australia[1], have developed media guidelines for safe reporting of suicide. These guidelines target media professionals and have been largely focused on traditional forms of news and print media, rather than the internet and social media. However, young people increasingly use social media platforms to discuss suicide in a number of ways. Strategies focused on involvement of professionals and on traditional forms of media are therefore less likely to be helpful for young people.

To date, there is a lack of evidence about safe and helpful online peer-to-peer communication about suicide, and there is little guidance available to help young people safely discuss suicide online. The aim of this project was to develop a set of evidence-informed guidelines that could help young people to communicate safely online about suicide.

1. Hunter Institute of Mental Health. Reporting suicide and mental Illness: A Mindframe resource for media professionals. Newcastle; 2014.

How the guidelines were developed:

The Delphi Consensus Method.

The #chatsafe guidelines were developed using the Delphi consensus methodology, that draws on expert opinions to identify best practice when evidence is lacking, and has been used in the development of guidelines for other mental health topics[2].

Our Delphi study consisted of two parts. The first was a systematic search of peer-reviewed and grey literature (e.g., websites and reports), in order to identify specific actions that young people could take when communicating online about suicide. These action statements were then entered into a questionnaire and their importance for inclusion in the guidelines was rated by two expert panels. The first panel included young people identified through Orygen, The National Centre of Excellence in Youth Mental Health's youth networks. The second panel consisted of suicide prevention experts, and media and communications professionals.

The role of the panel members was to rate each item according to how important they considered it to be for inclusion in the guidelines, across two rounds of questionnaires. Items that were endorsed as 'essential' or 'important', by at least 80% of both panels, were included in the final guidelines.

[2] Jorm AF. Using the Delphi expert consensus method in mental health research. The Australian and New Zealand journal of psychiatry. 2015;49(10):887-97.

Description of the guidelines

The #chatsafe guidelines provide tools and tips for young people to help them communicate safely online about suicide.

These guidelines consist of the following sections:

1. Before you post anything online about suicide;
2. Sharing your own thoughts, feelings or experience with suicidal behaviour online;
3. Communicating about someone you know who is affected by suicidal thoughts, feelings or behaviours;
4. Responding to someone who may be suicidal;
5. Memorial websites, pages and closed groups to honour the deceased.

Glossary of terms

Clickbait: content designed to encourage users to click on a link to a specific page or website.

Comment: refers to the process that allows users to respond or give feedback to a post.

Communicating about suicide: refers to any time that you are sharing, exchanging, or engaging with information, news or ideas about suicide. This includes creating your own posts; sharing someone else's post; sharing images, websites or links that might involve suicide content; or responding to someone who might be experiencing suicidal thoughts, feelings or behaviour.

Copycat suicide: occurs when exposure to suicide influences suicidal behaviour in another person. It can occur through direct exposure to suicidal content or indirectly through knowledge of another person's suicide.

Direct message: also known as a "DM", is a form of online communication sent between users privately on social media. It might also be referred to as "inboxing."

Emoji: refers to a small image or icon that can be used to express a reaction to a user's post. Some examples of emojis are below:

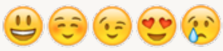

Links: refers to a hyperlink to another document, website or online content. It is often activated by clicking on a highlighted word or image.

Like: refers to a positive response that can be made to a post. Some online platforms may have a range of reactions that users can select when responding to posts. Facebook, for example, includes emoji reactions that can be used to respond to a post in various ways, including a 'sad face', 'angry face' and 'laughing face.'

Post: refers to the act of publishing your own original content online. This includes, but is not limited to, art, GIFs, images, links, memes, messages, photos, poetry, videos, Vines or vlogs to other websites/posts.

Private message: also known as a "PM". This term is often used interchangeably with "DM." Some online platforms, such as forums, use the term "PM".

Glossary of terms

Reply/respond: refers to the act of responding to a comment or direct message from another user online.

Safety plan: refers to a structured plan that has been developed with support from a health professional. It outlines actions, coping strategies, and supportive people that could assist you when you are experiencing suicidal thoughts, feelings or behaviour.

Screenshot: refers to the action of capturing content that is displayed on your screen and converting it to an image file that is stored on your personal computer or mobile device. Screenshots can also be referred to as "screen recording" or "screen grabs."

Share: refers to the act of re-publishing posts or content created by others on social media to friends or followers. This includes, but is not limited to, art, GIFs, images, links, memes, messages, photos, poetry, videos, Vines or vlogs to other websites/ posts.

Social media: refers to online platforms such as websites and mobile applications (apps) that allow users to share, communicate and create content that can be viewed by others. Popular examples include but are not limited to, Facebook, Instagram, Twitter, Tumblr, SnapChat, WeChat and Youtube.

Story/stories: refers to photos or videos that are taken throughout the day and published as a temporary post online. Some platforms, such as Snapchat, allow users to post stories that will disappear after 24 hours.

Trigger warning: also known as a 'Content notice'. Refers to a statement at the start of your post that alerts other users that the post may potentially contain distressing content. Trigger warnings can also be abbreviated to "TW" or "CN."

Trolling: negative content posted online with an intent to provoke or upset users.

Trusted adult or friend: refers to another person whom you feel comfortable talking to. It may be a person whom you feel is there for you and listens to you express your thoughts and feelings without judgment.

User: Refers to any individual who is exposed to content from social media platforms.

Viral: rapid online circulation of an image, video or other content from one user to another.

01

Before you post anything about suicide online

Before you communicate online about suicide, take some time to think about why you want to share this post. Reflect on how your post could affect other people and whether or not there is a different way to communicate this information in a way that is safer or more helpful.

It can also be helpful to be aware of some of the warning signs of suicide risk before you post online, as well as some of the suicide prevention resources offered by the social media or online platform you are using. For example, Facebook's Suicide Prevention Help Centre provides information on how to report suicide content to Facebook, as well as a number of resources and links to suicide helplines in Australia and internationally.

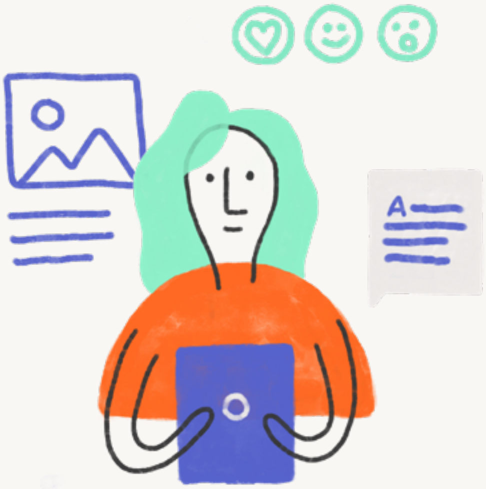

1.1

Remember that posts can go viral

Any image, photo, video or written post can quickly go viral online. If the post is inaccurate, stigmatising or unsafe, it could have a negative impact on yourself and others. It's important to be aware that once your post is published, you will have no control over who will see it or who will share it. It can be helpful to check the privacy of your profile or online account and remember that if your account is not set to 'private', anyone can access the content that you post.

1.2

The permanency of the internet: "Once posted, always posted"

It is possible that what you post or share online may be there forever. Even if you have sent a private message to a friend, or you have made a post that you later delete, it is possible that it will never be permanently erased. For example, other users could take screenshots of your messages and posts or they could create screen recordings of a live stream or story. As a consequence, your friends, parents, caregivers, work colleagues and current or future employers may later see it.

1.3

It can be helpful to monitor your post

Interacting with others through comments and posts is a key element of engaging with others online. If you have made a post that refers to suicide or suicidal behaviour, or you have shared or replied to a post that involves suicide-related content, it can be helpful to monitor your post regularly for unsafe or harmful comments (see below for examples of harmful comments).

If you do come across unsafe or harmful responses to your post, you should avoid arguing with other users in the comments section. Instead you could consider doing the following:

- Hide the user's post, if the platform allows it.
- Report unsafe content to the relevant social media suicide prevention help centre, if available.

Responding to someone who may be at risk of suicide can feel like a big responsibility. If you see a post that suggests someone may be at risk of suicide, you can find some tips on how to respond on page 28.

If you do come across suicidal content online, it's important that you also look after yourself. Some helpful tips for looking out for your own wellbeing are available on page 13.

Some examples of unsafe content

- Bullying
- Comments that include an invasion of privacy
- Graphic descriptions of suicide
- Graphic images of suicide
- Means or methods of suicide
- Plans of suicide (e.g., when or how)
- Spamming or repetitive content
- Statements that encourage people to take their own life
- Statements that appear to deliberately seek to trigger difficult or distressing emotions in other people
- Statements that include suicide pacts or suicide partners
- Statements that place blame or make others feel responsible for another person's safety
- Statements that provide vulnerable people information about how to end their life
- Suicide notes or goodbye notes
- Swearing
- Trolling
- Verbal attacks

1.4

When monitoring isn't an option

Sometimes you may not be able to monitor your own post regularly, you may no longer want to monitor your post, or you have shared content that can't be monitored on the social media platform you are using. If this is the case, in your original post, you could include the phone numbers or links to appropriate help services, such as helplines, local suicide prevention services, or local emergency services. Some Australian examples are provided on page 16.

1.5

Some tips on self-care

Whether you are an occasional or frequent user of social media, be aware that sometimes repeated exposure to negative content (e.g., conversations, images or videos about suicide) could impact upon your own wellbeing. It's also possible that a one-off post may trigger negative thoughts and feelings. It's important to have a plan in place in case you do feel upset or troubled by posts that you have shared or seen.
If you are feeling upset or overwhelmed, there are a few things that you could try:

- Talk to someone about how you are feeling.
- Take a break. This might include physically stepping away for a while, logging out of your social media accounts or engaging in a different activity that doesn't involve social media.
- Take control of the content that you see. For example, if you are going through a rough patch, you may want to minimise the amount of suicide-related content you are exposed to by hiding certain posts on your feed or unfollowing content that may cause you distress.

1.6

General safety tips for communicating about suicide online

There are many complex factors that lead to someone feeling suicidal, so it is important to communicate about suicide in a safe way. This is particularly important when communicating online, because the information you share can reach tens of thousands of people, extremely quickly. It is possible that some people, who already feel vulnerable, may engage in copycat suicide behaviour or may be negatively affected following exposure to suicide-related content online. Particularly when the content is extensive, exaggerates, or sensationalises suicide; repeats myths or misperceptions about suicide; or presents suicide in a positive or glorified way.

Pages 17-20 include some general tips for communicating about suicide. This includes some things that are unhelpful, as well as helpful alternatives that you could use.

1.7

Posting and sharing images, photos, or video content

Social media platforms often encourage the use of images, photos, videos and other multimedia. However, these can sometimes have unintended harmful consequences, particularly if they portray a person who has died by suicide.

Images, photos, video content or animations that depict a method of suicide or self-harm or the location of a suicide should be avoided. These can be upsetting to others who may be grieving and may lead to copycat behaviour by other people who are feeling vulnerable.

It is also recommended not to post or share images of people who have died by suicide looking dishevelled, threatening or clutching their head in distress. These images can reinforce negative stereotypes of a suicide and may be upsetting to others.

Australian suicide prevention resources available through different social media platforms

Facebook

Facebook Help Centre has a number of tools to help people who have come across suicide-related material. The Suicide Prevention Help Centre provides information on how to report suicide content to a trained member of their safety team who will identify the post and the location of the user. If necessary, they can contact emergency services to assist those at risk of suicide or self-harm. The Suicide Prevention Help Centre also provides information on country-specific suicide prevention helplines to assist people who may be experiencing suicidal thoughts, feelings or behaviour.

Instagram

Instagram Help Centre provides details to assist users to report content that suggests a person may be at risk of suicide or self-harm. Users can report content by:

1. Selecting ••• (on iOS) or ⋮ (on Android) above the post and tapping "Report";
2. Tap "It's inappropriate";
3. Selecting "Self injury";
4. Tapping "Report".

The Help Centre also provides links to suicide prevention websites an hotlines that can assist people during a suicidal crisis.

Snapchat

Snapchat Support Centre recommends users who are concerned about a fellow user encourage the person to seek help or consult with a professional service. If users don't feel comfortable engaging with the person who may be at risk of suicide, they can report a safety concern by:

1. Going to the snap you want to report;
2. Opening the snap and tapping the 🏳 button that appears in the bottom-left corner;
3. Select "More options"; Select "I'm worried that this Snapchatter might hurt himself/herself".

Twitter

Twitter Help Centre provides information on how to report self-harm and suicide-related content to a trained team devoted to responding to people who share content that suggests they may be at risk of self-harm or suicide. Information on how to recognise the signs of self-harm and suicide are provided, as well as an online form to alert the Twitter suicide prevention response team.

National phone and online support services available in Australia

e-headspace

Provides email, chat and phone counselling for young people aged between 12 and 25 years. eheadspace operates seven days a week, from 9:00am to 1:00am AEDST.

Website
https://www.eheadspace.org.au/

Phone
1800 650 890

Lifeline Australia

Provides free 24/7 online and phone personal crisis support and suicide prevention services to all Australians.

Website
https://www.lifeline.org.au/

Phone
13 11 14

Kids Helpline

Provides free and confidential 24/7 phone and online counselling for children and young people aged between five and 25 years.

Website
https://kidshelpline.com.au

Phone
1800 551 800

Suicide Call Back Service

Provides free 24/7 telephone, online, and video counselling and crisis support to all Australians affected by suicide.

Website
https://www.suicidecallbackservice.org.au/

Phone
1300 659 467

Language and safety tips for discussing suicide online

Unhelpful language

Don't use words that describe suicide as criminal or sinful. This may suggest to someone that what they are feeling is wrong or unacceptable, or make someone worry that they'll be judged if they ask for help.

Examples:

- Don't say "committed suicide".
- Don't say that suicide is a "solution" to problems, life stressors or mental health difficulties.

Don't use words that glamourise, romanticise, or make suicide seem appealing.

Examples:

- Don't share, quote or "like" the content of a suicide note or message.
- Don't post, share or include links to pro-suicide sites or forums. Don't provide information about suicide pacts.

Helpful alternatives

- Try to say the person "died by suicide".
- Indicate that suicide is complex and that many factors contribute to a person ending their life.
- Include messages of hope and recovery (e.g., you can post links to video's or poetry which has content about hope and recovery).
- Inform others that suicide can be prevented and include links to sources of help and websites that contain information about suicide prevention. Some examples include:
    - eheadspace
    - Kids Helpline
    - Lifeline
- Tell others who might be thinking about suicide where and how they could get help.
- Include information on factors that protect against suicide (e.g., engaging in meaningful activities and building and maintaining meaningful connections and relationships).
- Indicate suicide is preventable, help is available, treatment can be successful, and that recovery is possible.

Language and safety tips for discussing suicide online (continued)

Unhelpful language

Don't use words that trivialise or make suicide seem less complex than it really is.

Examples:

- Don't blame one event or imply the suicide was the result of a single cause, such as bullying or social media.
- Don't say that suicide is a solution to a problem, life stressors or mental health difficulties.

Don't sensationalise suicide.

Examples:

- Don't provide links to sensational "clickbait".

Helpful alternatives

- Try to say the person "died by suicide".
- Indicate that suicide is complex and that many factors contribute to a person ending their life.
- Include messages of hope and recovery (e.g., you can post links to video's or poetry which has content about hope and recovery).
- Inform others that suicide can be prevented and include links to sources of help and websites that contain information about suicide prevention. Some examples include:
    - eheadspace
    - Kids Helpline
    - Lifeline
- Tell others who might be thinking about suicide where and how they could get help.
- Include information on factors that protect against suicide (e.g., engaging in meaningful activities and building and maintaining meaningful connections and relationships).
- Indicate suicide is preventable, help is available, treatment can be successful, and that recovery is possible.

Language and safety tips for discussing suicide online (continued)

Unhelpful language

Don't use judgmental phrases which reinforce myths, stigma, stereotypes or suggest nothing can be done about suicide.

Examples:

- Don't say suicide is for "cowards", "a cry for help", "attention seeking" or "a selfish act".
- Don't use stigmatising words such as "crazy", "psycho", "nuts", "lunatic", "deranged", "defective", "insane" or "abnormal".
- Don't suggest that if someone wants to end their life then there is nothing you could do.

Don't provide detailed information about the actual suicide or suicide attempt.

Examples:

- Don't provide information about suicide methods, the location of a suicide. Don't acknowledge if that there have been a number of suicide acts at a particular location or "hotspot".

Helpful alternatives

- Try to say the person "died by suicide".
- Indicate that suicide is complex and that many factors contribute to a person ending their life.
- Include messages of hope and recovery (e.g., you can post links to video's or poetry which has content about hope and recovery).
- Inform others that suicide can be prevented and include links to sources of help and websites that contain information about suicide prevention. Some examples include:
  - eheadspace
  - Kids Helpline
  - Lifeline
- Tell others who might be thinking about suicide where and how they could get help.
- Include information on factors that protect against suicide (e.g., engaging in meaningful activities and building and maintaining meaningful connections and relationships).
- Indicate suicide is preventable, help is available, treatment can be successful, and that recovery is possible.

Language and safety tips for discussing suicide online (continued)

Unhelpful language

Don't describe suicide as a desirable outcome.

Examples:

- Don't use words or phrases such as "successful", "unsuccessful" or "failed attempt".
- Don't mention any positive consequences of suicidal behaviour that refer to suicide as a "relief", that the person is "finally at peace" or that suicide is "quick" or "painless".
- Don't say a suicide was "achieved", was "noble" or that it was a "brave act".

Helpful alternatives

- Try to say the person "died by suicide".
- Indicate that suicide is complex and that many factors contribute to a person ending their life.
- Include messages of hope and recovery (e.g., you can post links to video's or poetry which has content about hope and recovery).
- Inform others that suicide can be prevented and include links to sources of help and websites that contain information about suicide prevention. Some examples include:
    - eheadspace
    - Kids Helpline
    - Lifeline
- Tell others who might be thinking about suicide where and how they could get help.
- Include information on factors that protect against suicide (e.g., engaging in meaningful activities and building and maintaining meaningful connections and relationships).
- Indicate suicide is preventable, help is available, treatment can be successful, and that recovery is possible.

02

Sharing your own thoughts, feelings or experience with suicidal behaviour online

There may be times where you want to share your own experience with suicidal thoughts, feelings or behaviour. If you are currently experiencing suicidal thoughts, feelings or behaviours you should talk to a trusted adult or friend or reach out to a professional mental health service before posting online. If you have a 'Safety Plan', you can use the strategies outlined in it to help keep yourself safe. If you are feeling unsafe due to your own suicidal thoughts, feelings or behaviour you should contact your local emergency department by phone or go to emergency for assistance.

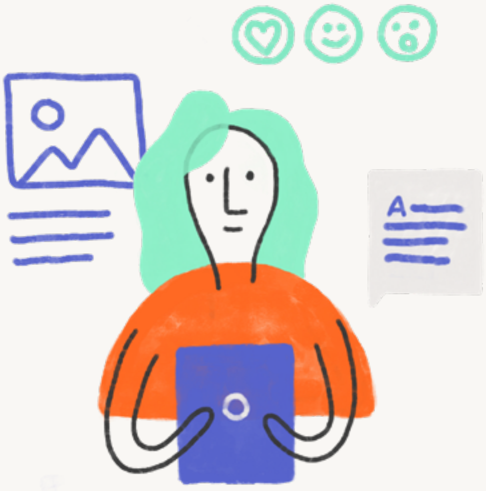

2.1 Your privacy

If you decide to post about your experience with current or previous suicidal thoughts, feelings or behaviour online, consider that other people in your life who don't know about your experiences might find this out as a result of your post. You may also receive a mixture of both positive and negative responses. Think about what aspects of your personal experience you wish to disclose and remember that you don't have to share everything. Only disclose the personal details you feel comfortable letting others know about and leave out any information you would not want everyone you have ever met, or will ever meet, to know about you.

Before you post about your own experience it can be helpful to ask yourself the following:

- How will sharing your experience make you feel? For example, if you think it could be distressing, you could ask a sibling, trusted adult or friend to review your post before you share it.
- What do you hope to achieve by sharing your experience? Do you want to raise awareness or are you looking for support? Are there more effective ways of achieving those things?
- Do you think you would benefit from seeking help about your current or previous experience with suicidal thoughts, feelings or behaviour? What resources, people or friends are available to help you?
- Who will see your post? For example, are you posting on a professional mental health site, an anonymous platform or a public platform? For example, if you are looking for professional help consider reaching out to a professional website.
- How might your post might affect your friends, family and peers?
- Will people who have read your post know how to help themselves or their loved ones after reading about your experience? If not, it could be helpful to provide some suggestions.

2.2

Sharing your experience in a safe and helpful way

You might find it helpful to have a plan in place about what to do if a person indicates that they are feeling distressed as a result of your post. For example, you could provide them with information outlined on page 16 or provide a link to a support service such as a suicide prevention or counselling helpline. If you include links to support services, they should be placed clearly at the beginning of your post and only include services that you know are reputable.

It can also be helpful to emphasise parts of your experience that demonstrate the importance of seeking help early and messages that reduce stigma and promote hope and recovery. Some examples include:

- The people, activities and actions that supported your recovery and how you coped.
- The positive experiences you had when seeking help.

2.3

The use of "trigger" or "content" warnings

If your post does include graphic or descriptive content or content that might be distressing to others, you should consider providing a trigger warning in your post. A trigger warning is a statement at the start of your post that alerts other users that the post may contain distressing content. The trigger warning should be positioned at the beginning of your post so readers can make an informed decision about whether or not they continue to read the post. It should also include a link or information on available support services.

If your post contains images, photos or videos, you should consider providing a trigger warning first and posting the content in the comment section or feed below so that other users can decide whether they want to see your post or not.

Remember to check your post for any unhelpful language, descriptions, images or graphic references to self-harm or suicide and remove them before you publish your post (see page 14 and pages 17-20 for some general tips).

03

Communicating about someone you know who is affected by suicidal thoughts, feelings or behaviour

Be aware that everyone is entitled to privacy, including those who have died by suicide. If you are concerned about someone who has experienced suicidal thoughts, feelings, or behaviour, do not post anything you would not say directly to them in-person. Where possible, ask for permission before posting or sharing content about another person and only share their personal information if you have permission to do so.

If you're concerned about someone because of the content of their post, talk to the person directly, either on or offline, before posting your concerns. It may also be helpful for you to inform a trusted adult or friend and, if available, seek professional advice.

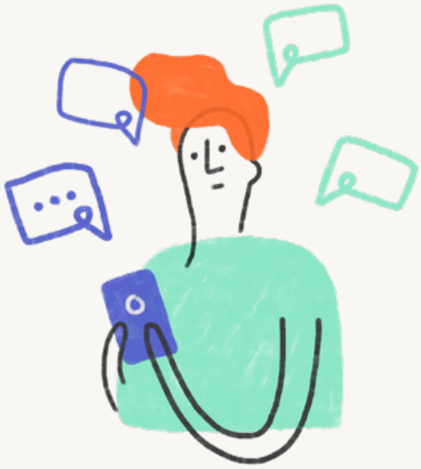

3.1

When someone has died by suicide

If you know someone who has died by suicide, be aware that their family members or friends might see your post about their loved one's death. They are likely to be grieving and struggling with a range of intense feelings, so it's important to be sensitive to those feelings and careful with the language you use.

If you are writing or sharing a post about someone who has died by suicide, only post what you know to be true and encourage others to do the same. You should avoid:

- Speculating about an individual's thoughts or feelings leading up to the suicide or about why the person took their life.
- Posting or sharing information that you are unsure of or that you know is inaccurate.

3.2

The suicide of celebrities and other public figures

Celebrity suicides often receive a lot of attention online. It is common for people to read and share stories from a number of different feeds and sources. Too much exposure to the suicide of a celebrity or other well-known public figure can be upsetting and may lead to copycat suicide. Therefore, when communicating about the suicide of a celebrity, the following is advised:

- Do not post or share content that speculates about the suicide of a celebrity before it has been confirmed by an official source (e.g., a well-known and reliable news website).
- Do not post or share too many posts about the celebrity suicide back to back, as too much exposure to this type of content can have a negative effect on vulnerable people.
- Avoid sharing content that discusses the celebrity suicide in an unsafe way. Where possible, share media articles that adhere to suicide prevention guidelines or the general tips outlined on pages 17-20.
- If you share a post on a celebrity suicide, you should also provide links to available support services.

04

Responding to someone who may be suicidal

Reaching out to others online can be an important source of affirmation, connection and support for many. Always take any content that suggests a person may be thinking about suicide seriously, but make sure you set boundaries about the type of support you are able to offer and when you are able to offer it.

Regardless of whether you think someone may be at immediate risk of suicide or not, you should put your own wellbeing first and do not put yourself in any physical danger.

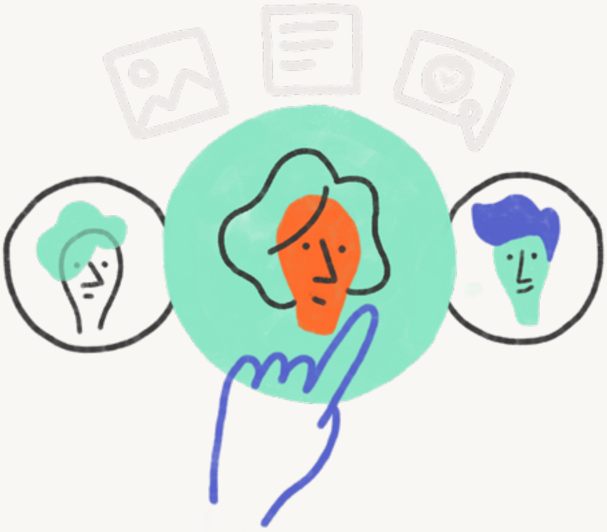

Before responding to someone who has indicated they may be at risk of suicide, ask yourself the following:

- How are you feeling?
- Are you able to provide support to the person if needed?
- Do you know where you could seek support and where you could refer the other person to for more comprehensive support?
- Is there someone else who is better placed to help?
- Will responding to the person make you feel unsafe or upset?
- Are you confident about how to deal with this situation?
- Do you understand your role and the limits of the support you could provide?

If you are concerned or do not feel comfortable responding to a post that suggests someone may be at risk of suicide, that is ok. Do not feel like it is your responsibility to reach out on your own. Instead you could do the following:

- Inform a trusted adult or friend.
- Seek professional advice (e.g., a phone or online service, or health professional).
- Report suicidal content to the relevant platform (e.g., Facebook Help Centre).

4.1

How to respond to someone who might be at risk of suicide

If you decide to respond to a person who may be at risk of suicide, let the person know that you care about them and respond without judgement, assumptions or interruptions. Let the person at risk explain their thoughts, feelings and experiences in their own words. When responding to someone who may be at risk of suicide, here are a few things that you could do:

- Always respond in private (e.g., through DM or PM), as the conversation may become upsetting or triggering for others.
- Look at the person's posts to acknowledge their feelings and specify exactly why you are worried about them (e.g., "from what you've posted it seems as though you are having a tough time").
- If the comment is on your own post, consider removing or hiding it, and providing them with support or help seeking options privately.

4.2

It's OK to ask someone "Are you thinking of suicide?"

Ask the person directly if they are thinking of suicide. Research has shown that there is no evidence that asking about suicide increases the likelihood of a person engaging in suicidal behaviour[3]. If you are worried or concerned that someone might be experiencing suicidal thoughts, feelings or behaviour, here are some questions that you could ask:

- "Are you thinking of suicide?"
- "Do you feel suicidal?"
- "Are you thinking of ending your life?"

You could also look at the person's posts to specify exactly why you are worried about them. For example, in a direct or private message you could say:

"I just wanted to check in, because you posted , and I am worried about you. It sounds like you might be feeling suicidal, is that the case?"

Don't let embarrassment or concern about offending or upsetting the person stop you from reaching out and offering help. There is no "perfect" or "right" thing to say to someone in these situations and it is better to make a supportive attempt to reach out than to make no attempt at all.

3. Blades CA, Stritzke WGK, Page AC, Brown JD. The benefits and risks of asking research participants about suicide: A meta-analysis of the impact of exposure to suicide-related content. Clinical Psychology Review. 2018;64:1-12.

4.3

If the person indicates they are not at immediate risk of suicide

If you contact the person and they indicate that they are not at immediate risk of suicide, reassure the person that support is available and encourage them to seek professional help. Some examples of things you could do are:

- Ask the person "do you want to talk about it?" or "what can I do to help?"
- Ask the person if they know where to get help.
- Ask the person if they would like the phone number or link to a local crisis support, mental health helpline or e-mental health service (e.g., e-headspace or Kids Helpline).
- If the person agrees, provide them with the phone number, links and relevant information to local support services.

If the person does not want help, respect what they tell you. If you feel comfortable, you could check in with the person to demonstrate that you care. For example, you could DM, text, or call them to offer support.

If they say “no”, it’s best not to pressure them. Don’t always expect a positive response and don’t take this personally; this person might be struggling to cope and may feel angry, upset or ambivalent. They may appreciate your help later, when they’re feeling better. If the person doesn’t want help, here are some helpful ways you can respond:

- Tell them “it’s okay if you don’t want to talk to me, but it is important that you talk to someone.”
- Report the post to the appropriate social media Safety Team or Help Centre (e.g., Facebook Help Centre).
- Follow up with the person at risk to check they are safe and can remain safe.

If at any time you think you might have said the wrong thing, don’t panic. Show that you care and that you can see that they are going through a tough time.

4.4

How to respond to a person at immediate risk of suicide or in the process of making a suicide attempt

Sometimes it will be clear that a person is at immediate risk of suicide or a person may be in the process of making a suicide attempt. If this occurs, you should:

- Encourage the person at risk to call 000
- Contact the person's family or someone in their social network who may be able to check on them.

If you cannot reach the person, or anyone in their family or social network, or the person refuses to call emergency services you should:

- Call emergency services on their behalf.

Once you have contacted emergency services, you should:

- Contact a trusted adult or friend and let them know what is happening.
- Report the post to the appropriate social media Safety Team or Help Centre (e.g., Facebook Help Centre) who can trace the address of the person posting and alert emergency services.

If you contact emergency services on the person's behalf, keep the person at risk informed about what is happening, unless this might increase the risk to them or someone else. If possible, try to maintain a conversation with the person until help arrives. Be prepared to provide emergency services with the following information:

- Your name and contact details.
- The fact that someone has told you online they have a plan to end their life or are in the process of making a suicide attempt.
- What the person online has said that indicates that they are at immediate risk of suicide.
- The date and time of their post.
- The person's identity and current location, if you know this information.

05

Memorial websites, pages and closed groups to honour the deceased

There may be times when you want to set up a website, page or closed group to memorialise a person who has died by suicide. Setting up a page or group to remember someone who has died can be a good way to share stories and receive and provide support. But there is also the potential that certain content could negatively impact the thoughts and feelings of others. For this reason, some concerns have been raised about the potential for copycat suicides. If you create a page or closed group to memorialise someone who has died by suicide, there are a few things that you can do to make the memorial page or group safe for others. These include:

- Monitoring comments for harmful or unsafe content (see page 12 for harmful content, and pages 17-20 for language tips).
- Deal with any disagreements or unsupportive comments quickly, as they can discourage people from sharing how they feel.
- Include a 'Terms of Use' that outlines the rules for participating in the online memorial page or group.

5.1

The terms of use for memorial pages or groups

The 'Terms of Use' states the purpose of the memorial page or group, and outlines safety rules for communicating about suicide on the platform. The 'Terms of Use' should be accessible by users via a link at the top of the page, and all potential members/followers of the account, website, page, or group should be asked to read and accept these before they are granted access to the memorial page or group. The 'Terms of Use' should:

- Encourage users to look after their own wellbeing, and to take a break if things are getting too much.
- Make it clear that discrimination of any kind is unacceptable.
- Make it clear that graphic details about the suicide are not allowed.
- Outline the approval process for comments and feedback from other users.
- Explain when and how users can report harmful suicidal content to the appropriate social media monitoring authority (e.g., Facebook Help Centre), or to the memorial page or group administrators.
- If you choose to accept reports yourself, then provide a 'Contact Form' or other contact method (e.g., email address) for users to report unsafe or concerning content.
- Explain how administrators will respond to comments that indicate that a person may be at risk of suicide.
- Explain that violation of the 'Terms of Use' will result in having the comment/s deleted.
- Outline the steps that will be taken to remove comments or users who violate the 'Terms of Use.'

If you, as the administrator, decide to suspend, delete or block a user, let the person know you are concerned about their behaviour and clearly and carefully explain the reasons why you are concerned to the user.

5.2

Responding to comments that indicate a person is struggling or distressed

There may be times when members of the memorial page indicate that they are struggling or are feeling distressed. People who have experienced the suicide of a close friend or loved one may experience different stages of grief at different periods of time and reach out to others on the memorial page for support themselves. In addition to the guidelines on responding to someone who may be at risk of suicide on pages 26-30, administrators could consider developing a standard template for responding to members in distress. These tips will help you create a template:

- Personalise the message by using the person's name.
- Express concern for the person (e.g., "I am worried about you, because of X").
- Adapt your message to the person's situation, and reflect the language they have used to describe their feelings.
- Encourage the person to seek help, but do not recommend a service if the person has indicated they did not find it helpful (e.g., they have had a bad experience with a particular helpline or professional). Encourage them to keep an open mind about what might help in the future.
- Adapt your message to accommodate any barriers they feel are stopping them seeking help (e.g., if they don't have mobile phone credit, recommend an accessible service that has a call back feature or online chat).
- Administrators should be mindful to minimise repeated use of templates if the person has reached out for support before.

Orygen
The National Centre of Excellence in Youth Mental Health

chatsafe
